# A nickel N-heterocyclic biscarbene complex derived from caffeine enhances fluconazole efficacy against *Nakaseomyces glabratus*

**DOI:** 10.1128/spectrum.00759-26

**Published:** 2026-09-02

**Authors:** Cláudia Malta-Luís, Giulia Romeo, Giulia Francescato, Carolina Mariano, Dalila Mil-Homens, Ana Petronilho, Catarina Pimentel

**Affiliations:** 1 Instituto de Tecnologia Química e Biológica António Xavier, Universidade Nova de Lisboa 98819, Oeiras, Portugal; Uniwersytet Jagiellonski w Krakowie, Kraków, Poland

**Keywords:** *Candida*, antifungal resistance, nickel, antifungals, metals, *Candida glabrata*

## Abstract

Invasive infections caused by *Candida* spp. are associated with high morbidity and mortality, particularly in immunocompromised patients, and are increasingly difficult to treat due to rising antifungal resistance. In this work, we further investigate the antifungal properties of a previously reported nickel N-heterocyclic biscarbene derived from caffeine (compound 5) and describe the synthesis of an analogous nickel N-heterocyclic biscarbene based on benzimidazole (compound 8), designed to evaluate the impact of the biscarbene and xanthine frameworks on activity and toxicity. Compound 5 displayed selective activity against *Nakaseomyces glabratus* (*Candida glabrata*), inhibited biofilm formation, and showed greater cellular accumulation in this species. In a *Galleria mellonella* infection model, compound 5 significantly reduced fungal burden while exhibiting lower cytotoxicity than the benzimidazole analog. Importantly, although compound 5 and fluconazole are individually fungistatic, their combination was fungicidal against *N. glabratus*. In the presence of compound 5, the minimal inhibitory concentration of fluconazole decreased against both a fluconazole-resistant petite mutant and a respiratory-competent *N. glabratus* strain evolved *in vitro* to fluconazole resistance. Compound 5 increased the frequency of petite mutants, suggesting an effect on mitochondrial function; however, its retained activity against these mutants and its synergism with fluconazole in the petite background indicate an additional mechanism of action. These findings identify compound 5 as a promising antifungal adjuvant.

**IMPORTANCE:**

Invasive infections caused by *N. glabratus* are increasingly hard to treat because this pathogen is often tolerant to azoles and is developing resistance to echinocandins, leaving clinicians with few effective options. Our work explores a nickel–caffeine complex that shows selective activity against *N. glabratus* and low toxicity, features that make it attractive as a promising candidate for further preclinical evaluation. Notably, this compound enhances the activity of fluconazole, turning a commonly used fungistatic drug into a fungicidal combination against both susceptible and resistant *N. glabratus*. By also affecting mitochondrial function yet remaining active in respiratory-deficient mutants, the compound appears to act through mechanisms distinct from existing antifungals. These properties suggest that metal-based xanthine complexes could be developed as adjuvants to enhance azole efficacy against difficult-to-treat *N. glabratus* infections, addressing an important priority identified for fungal pathogens.

## INTRODUCTION

Invasive fungal infections caused by *Candida* spp. are associated with high morbidity and mortality worldwide, especially among immunocompromised patients (1). To manage these infections, only a few antifungal classes, namely polyenes, azoles, and echinocandins, are available; however, the number of clinical strains resistant to them has increased over the years, resulting in infections that are difficult or sometimes impossible to treat (2, 3). Prompted by this scenario, the World Health Organization has published the first-ever fungal priority list, categorizing some *Candida* spp. into the “critical priority group,” and highlighting the urgent need for novel antifungals with distinct mechanisms of action (4, 5).

Although *Candida albicans* remains the most common cause of invasive candidiasis, non-*C*. *albicans* species are emerging as clinical challenges (1). Depending on the geographic area, *Nakaseomyces glabratus*, clinically known as *Candida glabrata* (6), is the second most frequent cause of invasive candidiasis in hospital settings. To overcome the intrinsically low susceptibility of *N. glabratus* to azoles (6–8), echinocandins are typically recommended as first-line treatment for invasive candidiasis caused by this species. Nevertheless, the incidence of *N. glabratus* clinical isolates resistant to this class of antifungals is also increasing, with approximately 14% of fluconazole-resistant isolates exhibiting resistance to at least one echinocandin (9–11).

Metal-based compounds have re-emerged as promising antifungal agents in response to current treatment challenges (12, 13). These compounds show stronger antifungal activity than organic molecules because of physicochemical properties uniquely conferred by the metals (12–14). In particular, nickel(II) has attracted attention because, in addition to its abundance and low cost compared to other transition metals, it exhibits versatile coordination chemistry and redox behavior, which allow the formation of diverse structures with multiple mechanisms of action (13, 15–17).

We have previously reported the synthesis and antifungal activity of several nickel complexes bearing N-heterocyclic carbene precursors (NHCs) derived from xanthines (18). Xanthines have been widely used as ligands in metal complexes, since they offer to the structure electronic flexibility, strong donor ability, and compatibility with a wide range of transition metals (19–21). Within that series, it was particularly noteworthy that the only biscarbene derivative (compound 5), based on caffeine, exhibited remarkably low *in vitro* toxicity compared to its monocarbene counterpart, while maintaining selectivity against *N. glabratus*.

In this work, the effect of introducing an additional carbene was examined through the synthesis of a different compound, benzimidazole-based N-heterocyclic biscarbene (compound 8), demonstrating that the presence of an extra carbene moiety alone is insufficient to reduce cytotoxicity. Although compound 8 exhibited greater *in vitro* activity than compound 5, this trend was not observed *in vivo*, where compound 5 showed superior efficacy in a *Galleria mellonella* model of invasive candidiasis caused by *N. glabratus*. The selectivity and antifungal activity of compound 5, together with its synergistic interaction with the most widely used azole, fluconazole, were further evaluated, highlighting its potential as an adjuvant in combination therapy for overcoming antifungal resistance.

## RESULTS

### Synthesis of compound 8

**Fig 1:**
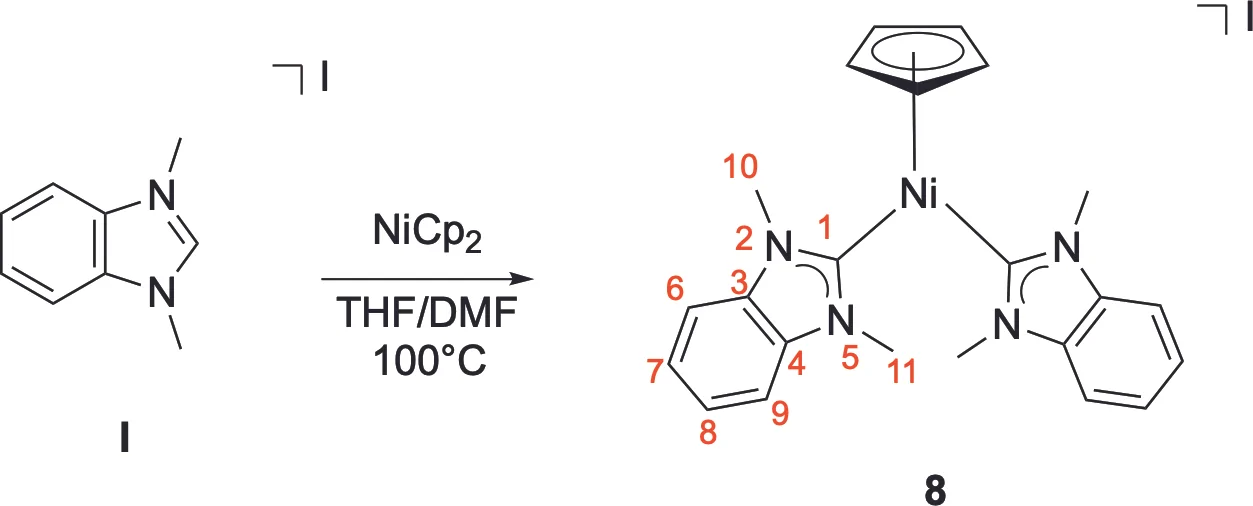
Synthesis of compound 8.

The benzimidazium salt I (22) was reacted with nickelocene at 100°C overnight, and after appropriate work-up, compound 8 was obtained in 22% yield (Fig. 1). Analysis of the solid by ^1^H NMR confirms the disappearance of H1 and the appearance of diagnostic multiplets at 7.60 and 7.30 ppm (Fig. S1). In ^13^C, the carbenic carbon resonates at 178.6 ppm, confirmed by 2D correlation experiments, a value similar to those of similar bis-NHCs reported for nickel (23).

**Fig 2:**
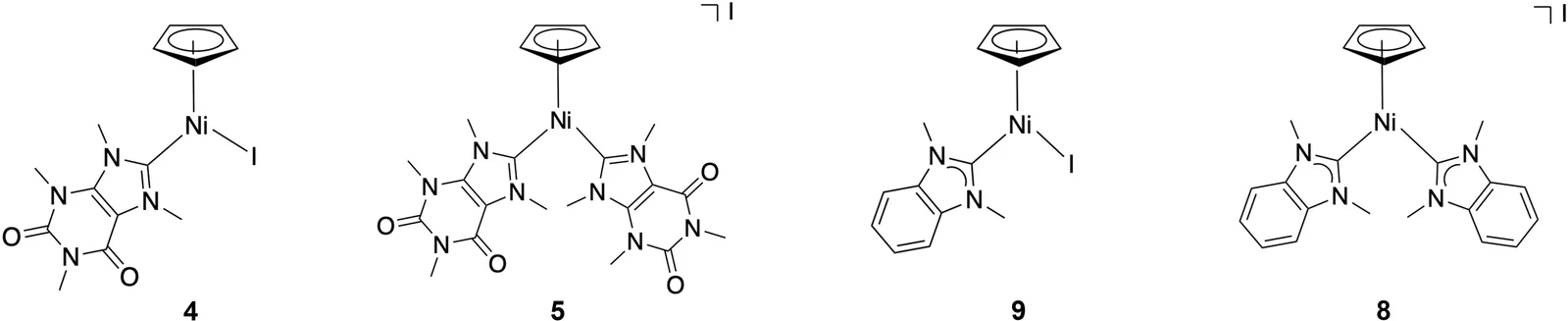
Molecular structure of mono- and bis-NHC complexes 4, 5, 8, and 9.

Compound 8 is characterized by a similar structure to that of compound 5 (Fig. 2), with both complexes being bis-NHCs based on fused heterocyclic rings. Their distinct nature stems from the type of N-heterocycle involved as a ligand. Compound 5 is based on the natural compound caffeine, a xanthine, for which biological relevance and role in medicinal applications are well documented.

### Effect of nickel complexes bearing N-heterocyclic biscarbene on antifungal activity and cytotoxicity

In a previous study, we showed that a biscarbene nickel complex of caffeine (compound 5), compared to the corresponding monocarbene complex (compound 4, Fig. 2), not only increased the specificity of the compound toward *N. glabratus* but also reduced its cytotoxicity in HeLa cells (18). Therefore, we assessed whether the same effect could be observed with the newly synthesized nickel complex of an N-heterocyclic biscarbene from a different chemical family, benzimidazole (compound 8, Fig. 2). The minimal inhibitory concentration (MIC) of compounds 5 and 8 against the two most prevalent *Candida* spp. associated with invasive candidiasis, *C. albicans* and *N. glabratus*, was determined according to the Clinical and Laboratory Standards Institute (CLSI) guidelines for yeast (24). As previously shown, compound 5 is highly selective for *N. glabratus*, exhibiting MICs 16-fold lower than those for *C. albicans* (Table 1 [18]). Compound 8 is more effective against both species (lower MICs) than compound 5 (Table 1) or its monocarbene counterpart (75 μM for *N. glabratus* and 156 μM for *C. albicans*; compound 9 in Fig. 2 and [18]), but selectivity for *N. glabratus* was not observed.

**TABLE 1:** Minimal inhibitory concentration (MIC) of 5 and 8 against *Candida albicans* and *Nakaseomyces glabratus*

Yeast species	MIC (μM)	
	5	8
*Candida albicans*	1,250	4.9
*Nakaseomyces glabratus*	78.1	2.4

**Fig 3:**
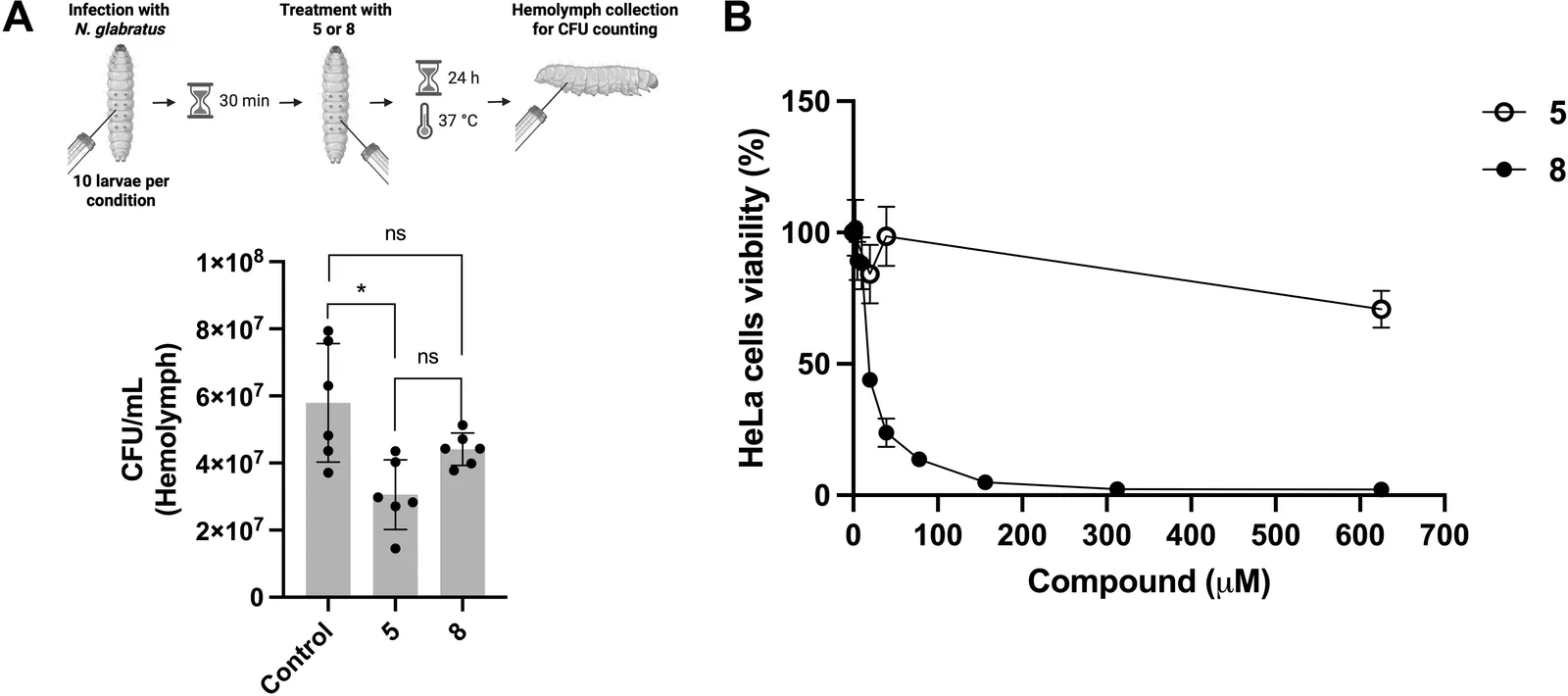
Compound 8 shows reduced *in vivo* activity against *N. glabratus* and higher cytotoxicity compared with compound 5. (**A**) Fungal burden in the hemolymph of infected *G. mellonella* treated with PBS (control), 312.5 μM of compound 5 (5), or 312.5 μM of compound 8 (8). Hemolymph was collected from six infected larvae per condition, after 24 h, and plated on yeast peptone dextrose (YPD) agar plates for colony-forming unit (CFU) counting. Statistical significance was assessed using one-way analysis of variance with Tukey’s HSD post hoc test (*P ≤ 0.05). (**B**) Cellular viability was evaluated using the 3-(4,5-dimethylthiazol-2-yl)−2,5-diphenyltetrazolium bromide assay, and the values are expressed as the percentage of viability relative to the untreated control.

Despite its potent *in vitro* activity, compound 8 showed reduced efficacy *in vivo* in the *G*. *mellonella* model compared with compound 5 (Fig. 3A). Administration of 312.5 µM of compound 8 following *N. glabratus* infection did not reduce fungal burden in the larvae hemolymph, whereas treatment with 312.5 µM of compound 5 led to a significant decrease. In addition, compound 8 remained highly toxic to HeLa cells (Fig. 3B). Based on its promising *in vivo* activity and lower toxicity, compound 5 was selected for further biological characterization.

### The caffeine-based N-heterocyclic biscarbene (compound 5) accumulates to higher levels in *N. glabratus* than in *C. albicans*

**Fig 4:**
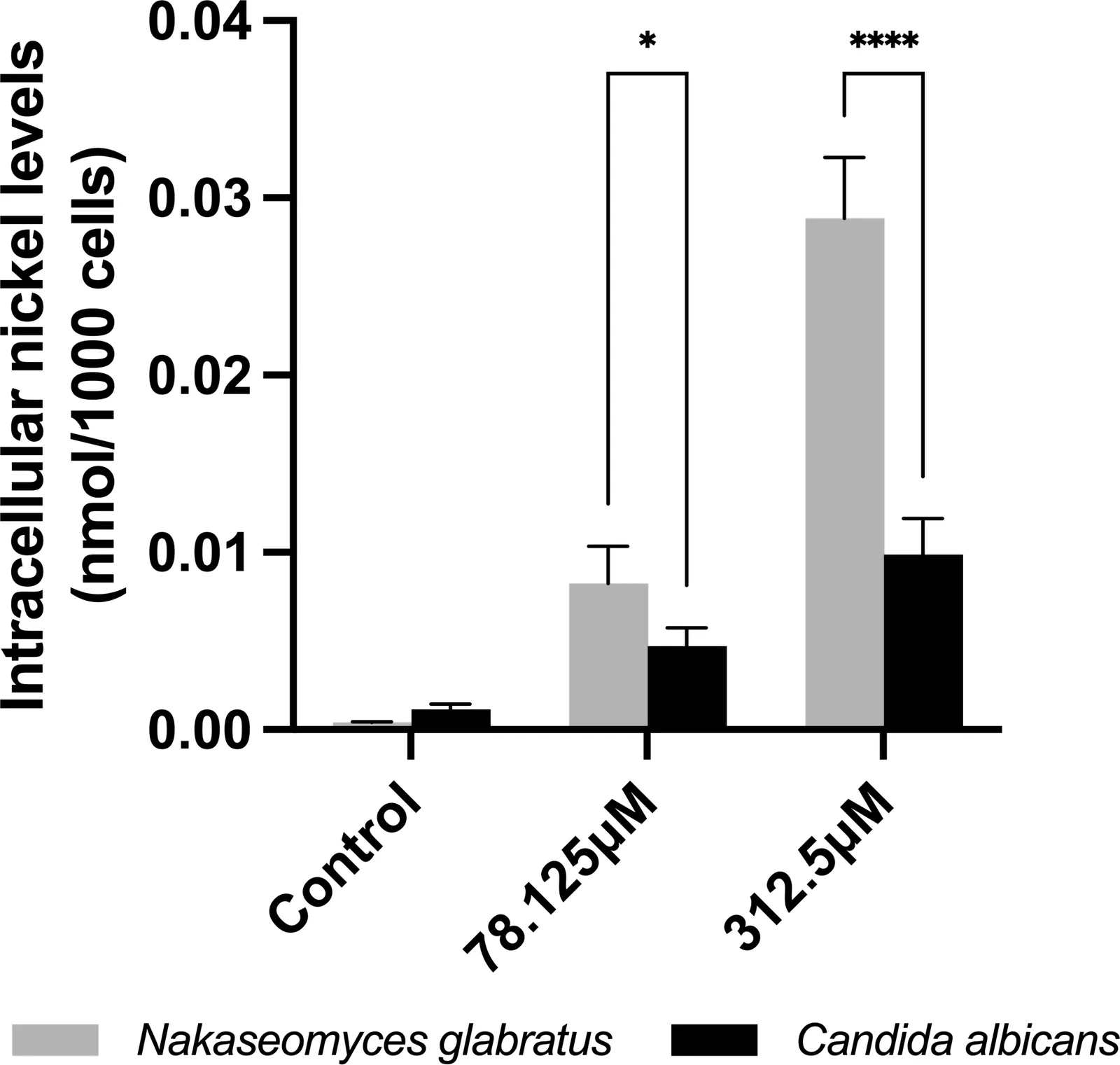
Higher intracellular levels of nickel after treatment with five were observed in *Nakaseomyces glabratus*. The nickel content of *N. glabratus* cells, either untreated (control) or treated with the indicated concentrations of 5, was quantified by ICP-AES. Data represent the mean ± standard deviation of four biological replicates. Statistical significance was assessed using two-way analysis of variance with Tukey’s HSD post hoc test (*P ≤ 0.05 and ****P ≤ 0.0001).

To investigate the specificity of compound 5 toward *N. glabratus*, we quantified the nickel content of *C. albicans* and *N. glabratus* cells exposed to either 78.1 μM or 312.5 μM of compound 5 (corresponding to the MIC and 4× MIC for *N. glabratus*, respectively) using inductively coupled plasma atomic emission spectroscopy (ICP-AES). At both concentrations, compound 5 accumulated to higher intracellular levels in *N. glabratus* than in *C. albicans* cells (Fig. 4), suggesting that its higher activity against *N. glabratus* may be linked to enhanced cellular uptake in this species.

The spectrum of activity of compound 5 was also evaluated against other clinically relevant *Candida* spp., included in the WHO fungal priority pathogens list, as well as against the baker’s yeast *Saccharomyces cerevisiae* (5). Consistent with its marked selectivity, compound 5 displayed activity against the phylogenetically related yeasts *N. glabratus* and *S. cerevisiae* and showed poor or no activity against other *Candida* spp. within the tested concentration range (Table 2). Importantly, compound 5 was equally effective against a fluconazole-resistant *N. glabratus* strain.

**TABLE 2:** Minimal inhibitory concentration (MIC) of 5 against medically relevant *Candida* spp

Yeast species	MIC (μM)
*Candida albicans*	1,250
*Nakaseomyces glabratus*	78.1
*Candida krusei*	>2,500
*Candida tropicalis*	>2,500
*Candida parapsilosis*	>2,500
*Nakaseomyces glabratus* resistant to fluconazole^*a*^ *Saccharomyces cerevisiae*	78.1 156.3

^*a*^ MIC_fluc_ = 209 µM.

### Compound 5 acts synergistically with the antifungal fluconazole

A current trend in antifungal therapy research is the combination of antifungal agents with new or existing compounds that can act synergistically to improve antifungal potency and reduce the risk of resistance (25–27). Within this context, the possible synergistic effect of compound 5 with fluconazole, one of the most prescribed antifungal drugs worldwide, was tested.

**Fig 5:**
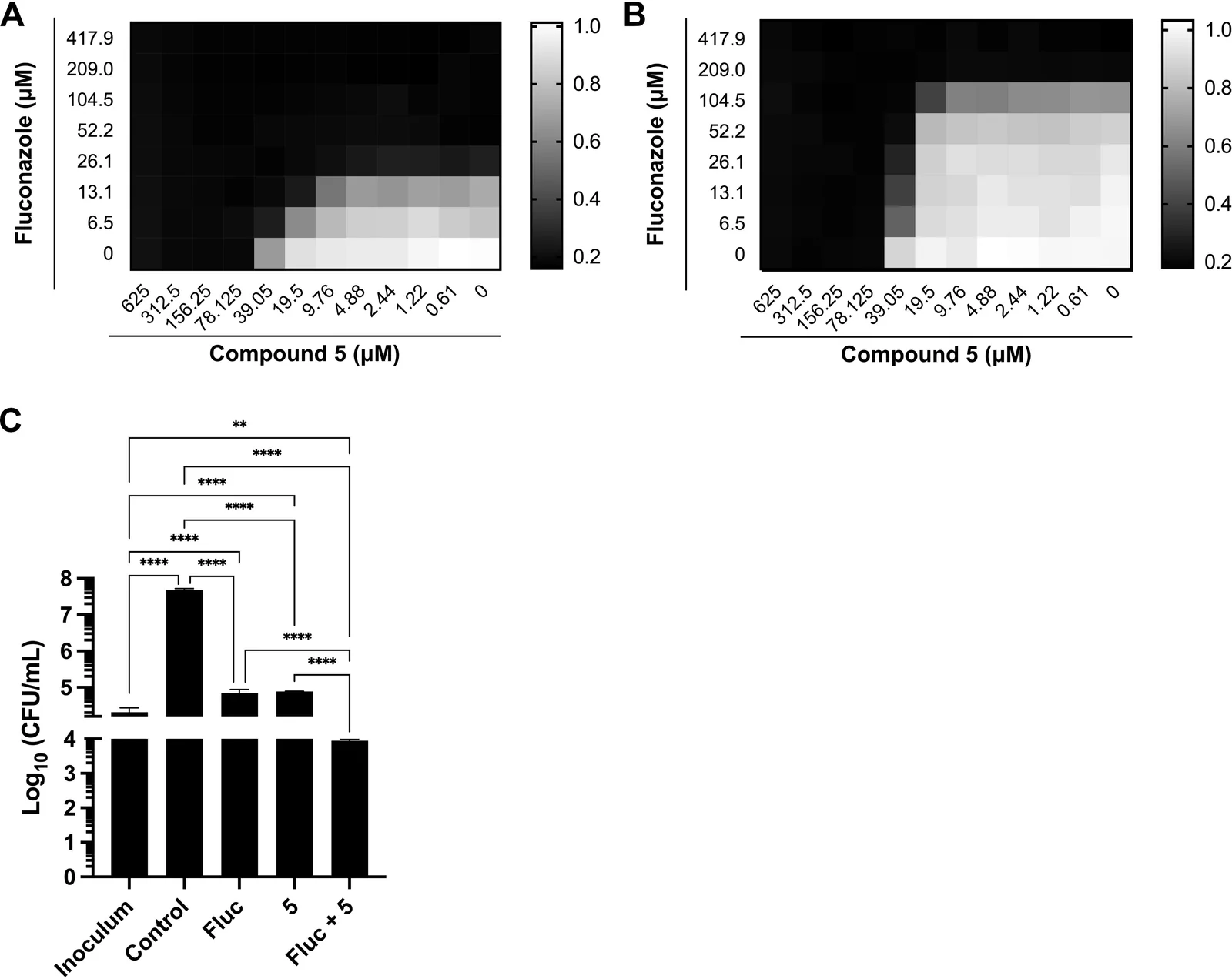
Synergistic interaction between compound 5 and fluconazole. Checkerboard assays were performed to evaluate the growth of (**A**) *N. glabratus* wild-type strain and (**B**) a fluconazole-resistant *N. glabratus* strain. After 24 h, OD_600_ was measured, values were normalized to untreated controls (left panels), and 5 μL of each culture was spotted onto YPD agar plates (right panels). (**C**) *N. glabratus* cell viability was further assessed by CFU counting of the initial inoculum (Inoculum), after 24 h of growth (control), or following treatment with 78.1 μM of 5 (5), 52.2 μM of fluconazole (Fluc), or their combination (Fluc + 5). Results are expressed as Log_10_(CFU/mL) and represent the mean ± standard deviation of three biological replicates. Statistical differences were determined using one-way analysis of variance on Log_10_-transformed CFU/mL values (**P ≤ 0.01 and ****P ≤ 0.0001). The regions corresponding to concentrations at which the combination is more effective than the individual drugs alone are delineated by the green line.

Using a checkerboard assay, we evaluated the growth of a wild-type *N. glabratus* strain (Fig. 5A) and a laboratory-evolved fluconazole-resistant *N. glabratus* strain (Fig. 5B) in response to a combination of twofold serial dilutions of compound 5 (0.6–625 μM) and fluconazole (6.5–417.9 μM). After 24 h of growth, the wild-type strain showed a marked decrease in cell density when treated with a combination of compound 5 (9.8–39 μM) and fluconazole (6.5 μM), compared with equivalent concentrations of each drug alone (Fig. 5A). A similar pattern was observed in the fluconazole-resistant strain (Fig. 5B). Determination of the fractional inhibitory concentration index (ΣFIC) clearly indicated a synergistic interaction between compound 5 and fluconazole (Table 3). The effect appears to be fluconazole specific, as it was not observed with representatives of other antifungal classes or with other triazoles (Fig. S3). Most importantly, although compound 5 (78.1 μM) and fluconazole (52.2 μM) exhibited fungistatic activity, reducing growth relative to the no-drug control without affecting cell viability compared with the initial inoculum, their combined treatment was fungicidal, as determined by colony-forming unit (CFU) counting (Fig. 5C). Notably, no toxicity was observed for the combination of fluconazole and compound 5 over a wide range of concentrations (Fig. S4).

**TABLE 3:** Antifungal susceptibility testing and drug interaction evaluation in fluconazole-susceptible and fluconazole-resistant *N. glabratus* strains

Yeast species	Standard MIC (μM)		Combination MIC (μM)		∑FIC	Interaction
	5	Fluconazole	5	Fluconazole		
*N. glabratus* ATCC2001	78.1	26.1	39	13.1	0.5	Synergy
*N. glabratus* resistant to fluconazole	78.1	209	39	104.5	0.5	Synergy
Compound 5-induced petite *N. glabratus* mutant	78.1	1,671.6	39	835.8	0.031	Synergy

**Fig 6:**
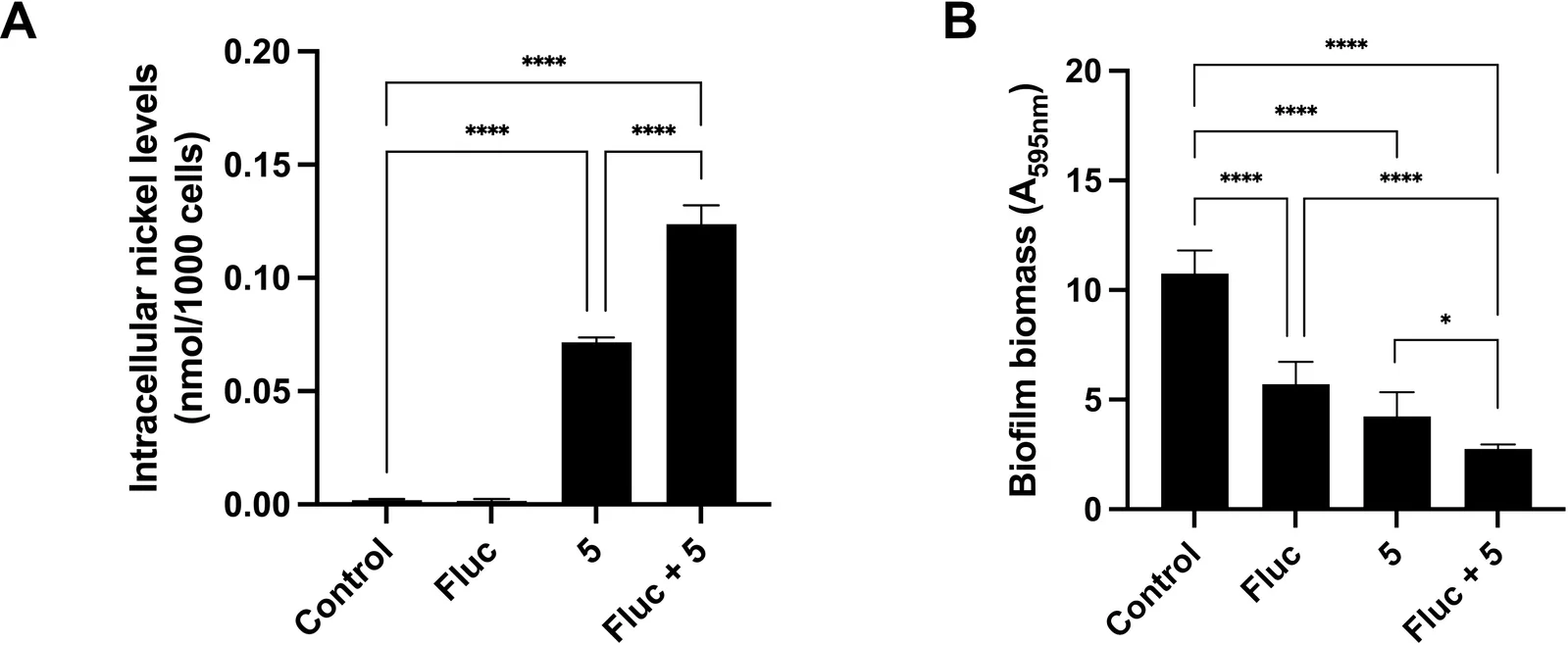
Combining compound 5 with fluconazole increases intracellular nickel levels and enhances biofilm inhibition. (**A**) Intracellular nickel content of *N. glabratus* cells, either untreated (control) or treated with 78.1 μM of compound 5 (5), 52.2 μM of fluconazole (Fluc), or their combination (Fluc + 5), was quantified by ICP-AES. Data represent the mean ± standard deviation of four biological replicates. Statistical significance was assessed using one-way analysis of variance (ANOVA) with Tukey’s HSD post hoc test (****P ≤ 0.0001). (**B**) Biofilm formation of *N. glabratus* either left untreated (control) or treated with 78.1 μM of compound 5 (5), 52.2 μM of fluconazole (Fluc), or the corresponding concentrations of both (Fluc + 5). Biofilm mass was quantified using the crystal violet staining method. Values represent the mean of eight replicates. Statistical significance was assessed using one-way ANOVA with Tukey’s HSD post hoc test (*P ≤ 0.05, **P ≤ 0.01, and ****P ≤ 0.0001).

To elucidate this synergistic effect, intracellular nickel levels were quantified by ICP-AES in *N. glabratus* cells co-treated with compound 5 and fluconazole. The combination resulted in a further increase in intracellular nickel content (Fig. 6A), suggesting that the observed synergy likely arises from enhanced cellular uptake of compound 5, possibly due to fluconazole-induced alterations in sterol levels (28).

One key virulence factor of *Candida* spp. is their ability to form biofilms on biotic and abiotic surfaces, contributing to persistent infections and increasing the risk of resistance development (29). Because caffeine has been reported to inhibit the formation of *C. albicans* biofilms (30), the impact of compound 5, a caffeine derivative, alone and in combination with fluconazole, on *N. glabratus* biofilm formation was evaluated using the crystal violet staining method. Compound 5 strongly impaired biofilm development, and this effect was even more pronounced when combined with fluconazole (Fig. 6B).

### Treatment of *N. glabratus* with compound 5 induces petite mutants

During cell viability assays (Fig. 5), we observed that treatment with compound 5, either alone or in combination with fluconazole, led to the appearance of a subpopulation of slow-growing colonies, which was particularly striking under co-treatment conditions. These colonies were smaller and became visible to the naked eye only after an additional 24 h of incubation, consistent with the characteristics of petite mutants, which arise following partial or complete loss of mitochondrial function in yeast (31, 32).

**Fig 7:**
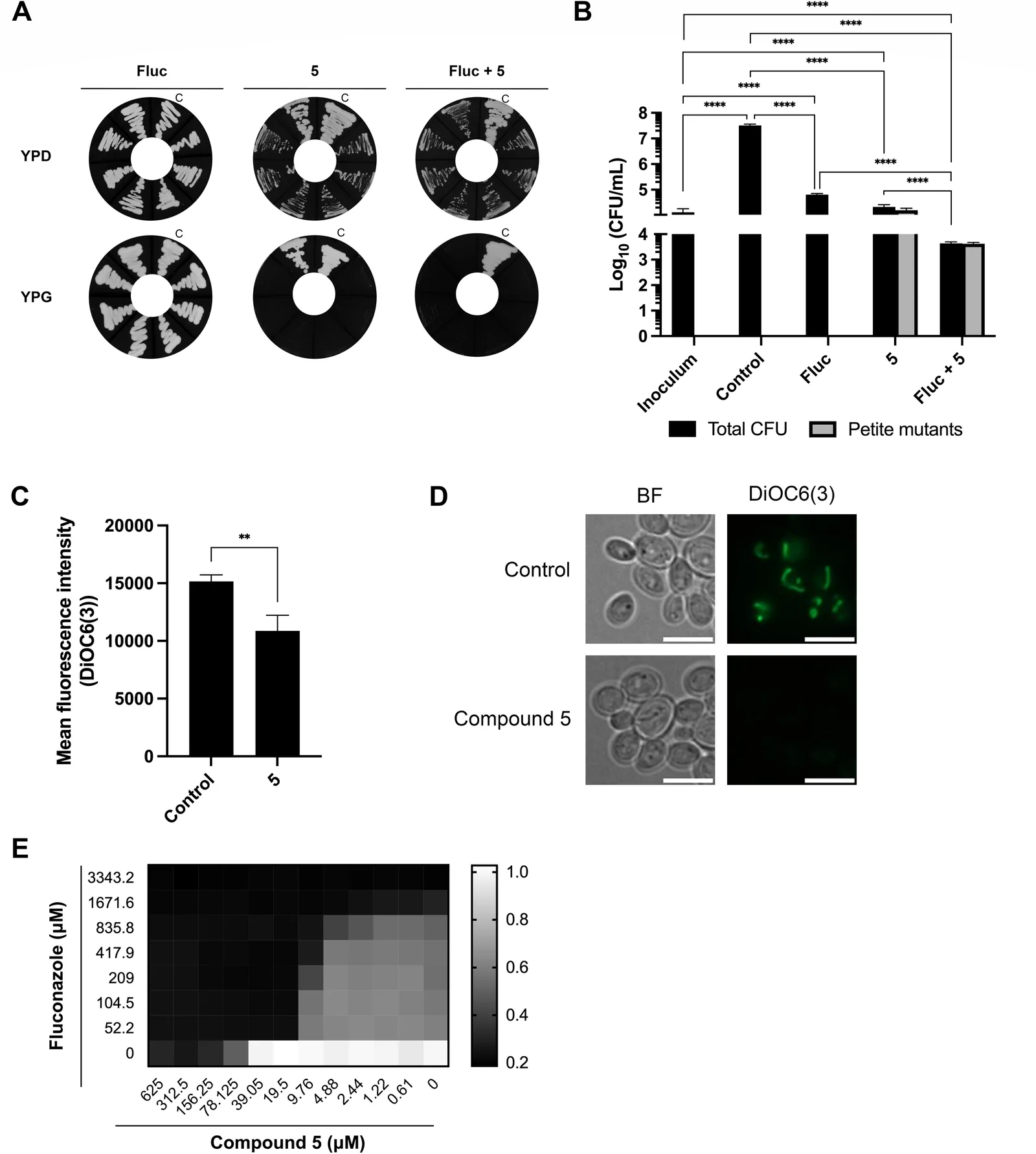
Compound 5 induces petite mutants. (**A**) Representative growth of slow-growing, small colonies on YPD and YPG agar plates. C-normal growing colony. (**B**) Quantification of petite mutants. *N. glabratus* cultures were treated for 24 h with 78.1 μM compound 5 (5), 52.2 μM of fluconazole (Fluc), or their combination (Fluc + 5). Statistical differences were determined using one-way analysis of variance on Log_10_-transformed CFU/mL values (****P ≤ 0.0001). (**C**) Changes in mitochondrial membrane potential in *N. glabratus* cells, untreated (control) or treated with 78.1 μM of compound 5, quantified using the fluorescent dye DiOC6(3). Values are the mean of three biological replicates. Statistical significance was assessed using Student’s *t*-test (**P < 0.01). (**D**) Confocal microscopy images of *N. glabratus* cells either left untreated (control) or treated with 78.1 μM of compound 5, stained with DiOC6(3). The scale bar represents 5 μm. (**E**) Checkerboard assays using a compound 5-induced petite mutant strain. After 24 h, OD_600_ was measured, and values were normalized to untreated controls (left panel), and 5 μL of each culture was spotted onto YPD agar plates (right panel). The regions corresponding to concentrations at which the combination is more effective than the individual drugs alone are delineated by the green line.

Since petite mutants are respiratory deficient and cannot grow on non-fermentable carbon sources (33), we randomly select slow-growing, small colonies and subculture them on YPD and YPG agar plates containing glucose or glycerol, respectively (Fig. 7A). The inability of some colonies to grow on YPG confirmed their respiratory deficient, petite nature. The formation of petite mutants was quantified by plating equal volumes of *N. glabratus* cultures, treated for 24 h with 78.1 μM of compound 5, 52.2 μM of fluconazole, or their combination, onto YPD and YPG agar plates. CFUs were counted after 24 h and 48 h, and the difference between YPD and YPG colony numbers was used to determine the number of petite mutants. While no petite mutants were observed during the growth period with fluconazole alone, approximately 70% of colonies exhibited respiratory deficiencies following treatment with compound 5, and 100% displayed this phenotype after co-treatment with both compounds (Fig. 7B). In addition, we assessed the mitochondrial membrane potential of yeast cells left untreated or treated with compound 5 using the fluorescent probe 3,3′-dihexyloxacarbocyanine iodide (DiOC6[3]; Fig. 7C,D). Treatment with compound 5 led to a decrease in DiOC6(3) fluorescence, indicating a loss of mitochondrial membrane potential and suggesting mitochondrial dysfunction. This finding is consistent with the ability of compound 5 to induce the formation of petite mutants, further supporting an adverse effect on mitochondrial function.

Petite mutants are well known to be resistant to fluconazole (34), and those induced by compound 5 exhibit an MIC of 1,672 μM, which is approximately 128-fold higher than that of the wild-type strain. We therefore questioned whether the synergy observed between compound 5 and fluconazole would be maintained in petite mutants. Checkerboard analysis using a compound 5-induced petite mutant demonstrated that the synergistic effect remained evident (Fig. 7E), indicating that the interaction is not abolished by the petite phenotype.

## DISCUSSION

The increasing number of *Candida* clinical isolates resistant to one or more antifungal agents, together with the limited pipeline of new antifungals, contributes to rising mortality rates, particularly among critically ill patients, underscoring the urgent need for novel antifungal therapies (1, 2, 35). In response to this clinical challenge, metal-based compounds have emerged as promising antifungal candidates (12, 13). Among transition metals, nickel has been highlighted in several reports as a metal of interest (13, 18, 21, 36–40). Structurally, most nickel(II) complexes described to date are Schiff-base ligands, leaving N-heterocyclic carbene-based nickel complexes largely underexplored.

We have previously identified compound 5, a nickel(II) N-heterocyclic biscarbene complex, as exhibiting selective activity against *N. glabratus* and low cytotoxicity (18). Because the corresponding monocarbene was highly toxic to mammalian cells and less effective, we hypothesized that the biscarbene framework might confer an advantage. However, this was not the case for a nickel(II) biscarbene of benzimidazole (compound 8). Although compound 8 showed greater *in vitro* antifungal activity than compound 5 and its monocarbene counterpart (18), it also exhibited higher *in vitro* toxicity and lower *in vivo* efficacy. *In vitro* potency does not necessarily translate into *in vivo* efficacy, possibly due to physicochemical constraints such as lipophilicity. Compound 8 is more lipophilic than compound 5 (logP 6.7 versus 1.4—theoretical calculation using MarvinSketch from Chemaxon). In a study of fenarimol analogs, compounds with lower lipophilicity were significantly more effective in increasing *G. mellonella* survival, whereas more lipophilic analogs showed reduced efficacy despite comparable *in vitro* activity, suggesting that high lipophilicity negatively affects *in vivo* performance in this model (41).

Compound 5 was selective for *N. glabratus* and *S. cerevisiae* but showed limited activity against other yeast species. *N. glabratus* is more closely related to *S. cerevisiae* than to other *Candida* spp., sharing a common ancestor that underwent whole-genome duplication (42, 43). Therefore, a possible explanation is that uptake of compound 5 is mediated by a transporter that is specific to these species, exhibits higher affinity for compound 5, or is overexpressed in these yeasts.

An interesting finding of this study is the induction of respiratory-deficient (petite) mutants by compound 5. Petite mutants typically appear when cells have undergone total or partial loss of mitochondrial DNA (mtDNA) (32, 33), often caused by oxidative stress (44). Xanthine-derived NHC complexes have mainly been studied for their anticancer activity, and some reports suggest oxidative stress-related mechanisms for selected complexes (45–47). However, under our experimental conditions, we did not detect any increase in reactive oxygen species (ROS) after exposing *N. glabratus* to compound 5 (Fig. S5), making ROS induction an unlikely mechanism for this compound. Another possible route for petite formation, observed with DNA intercalating agents such as ethidium bromide, is the binding of the drug to mtDNA, which leads to the inhibition of replication and the induction of excessive DNA repair, ultimately resulting in mtDNA deletions (48). In *S. cerevisiae*, caffeine has been shown to be a petite-inducing agent (49, 50). Wolf et al. (49) proposed that this phenotype may arise from binding of caffeine to mtDNA, either by stacking externally on terminal purine bases or by intercalating between purine bases (51), leading to mispairing and ultimately to loss of mtDNA. In this context, since compound 5 is a caffeine derivative, a similar mechanism cannot be excluded.

The fact that compound 5 induces petite mutants was surprising in the context of its synergism with fluconazole, as in *N. glabratus*, petite mutants are well documented to exhibit reduced susceptibility to fluconazole (34, 52, 53). In such cases, resistance has been attributed to mitochondrial dysfunction-driven retrograde signaling, which results in the constitutive upregulation of ATP-binding cassette drug efflux pumps such as Cdr1 and Cdr2 (54, 55). One possible explanation for the observed synergism is that additional mutations or cellular alterations induced by compound 5 contribute to its antifungal activity. Further support for this hypothesis comes from two observations: (i) the synergism between fluconazole and compound 5 remains evident in petite mutants, and (ii) the proportion of petite cells is markedly reduced upon co-treatment with fluconazole compared to treatment with compound 5 alone. Together, these findings reconcile the apparent paradox whereby compound 5 induces fluconazole-resistant petite mutants yet acts synergistically with fluconazole. A similar mechanism may apply to caffeine, as it has been reported to reduce the proportion of petite mutants when combined with intercalating agents such as ethidium bromide (56, 57).

Although fluconazole did not induce petite mutants under our treatment conditions, it is well known to promote this phenotype *in vitro*, and petite clinical strains have been isolated from fluconazole-treated patients (34, 58, 59). In such cases, compound 5 may provide a promising co-treatment strategy to counteract the resistance mechanism triggered by these mutants and thereby enhance the therapeutic efficacy of fluconazole.

## MATERIALS AND METHODS

### Synthesis of compounds 5 and 8

The synthesis of compound 5 was previously described in (18). Synthesis of compound 8: A Schlenk tube was charged with dimethylbenzimidazole (50 mg, 0.18 mmol) and nickelocene (34 mg, 0.18). After the addition of dimethylformamide (DMF) (1.8 mL), the reaction was heated at 100°C and stirred overnight. After cooling, the solvent was concentrated under vacuum and then Et_2_O was added to induce precipitation. The solid was separated by filtration, washed with Et_2_O, and dried, yielding the desired compound as a brown solid in 22% yield (11 mg). ^1^H NMR (400 MHz, DMSO): δ ^1^H NMR (400 MHz, DMSO): δ 7.61–7.58 (m, 4H, H7–H8 Ar, ^3^J_H7–H6_ = ^3^J_H8–H9_ ≈ 6.0 Hz, ^4^J_H7–H9_ = ^4^J_H8–H6_ ≈ 3.1 Hz); 7.31–7.28 (m, 4H, H6–H9 Ar, ^3^J_H6–H7_ = ^3^J_H9–H8_ ≈ 6.0 Hz, ^4^J_H6–H8_ = ^4^J_H9–H7_ ≈ 3.1 Hz); 5.71 (s, 5H, C_5_H_5_); 4.23 (s, 12H, N2-CH_3_, N5-CH_3_) ppm. ^13^C {^1^H} NMR (100 MHz, DMSO): δ 178.6 (C-1); 135.3 (C-3 and C-4); 122.9 (C-6 and C-9); 110.5 (C-7 and C-8); 92.4 (C_5_H_5_); 35.8 (N2-CH_3_, N5-CH_3_) ppm. HRMS-ESI (m/z): calcd: 415.1433; found: 415.1420.

### Strains and growth conditions

The *Candida* spp. used in this study are listed in Table 4. Strains were maintained on Sabouraud Dextrose Agar plates. Unless otherwise indicated, all assays were performed in Roswell Park Memorial Institute 1640 (RPMI-1640) medium with 0.165 M of 3-(N-Morpholino)propanesulfonic acid (MOPS) adjusted to pH 7.0. For ICP-AES assays, strains were grown in SC medium pH 5.5 (0.77 g/L complete supplement mixture; 1.7 g/L yeast nitrogen base without amino acids and ammonium sulfate; 5.4 g/L ammonium sulfate; 2% glucose). The wild-type (*N. glabratus* ATCC) and petite mutant strains were maintained in YPD medium (1% yeast extract, 2% peptone, 2% glucose, and 2% agar). Cell viability was determined by counting CFUs. To this end, cultures were serially diluted in PBS and seeded onto YPD agar plates. The presence of petite mutants was examined on YPG agar plates (1% yeast extract, 2% peptone, 2% glycerol, and 2% agar), and growth was recorded after 72 h of incubation at 37 °C.

**TABLE 4:** Yeast species used in this study

Yeast species	Origin
*Candida albicans* (SC5314)	ATCC
*Nakaseomyces glabratus* (ATCC 2001)	ATCC
*Candida krusei* (ATCC6258)	ATCC
*Candida tropicalis* (ATCC 750)	ATCC
*Candida parapsilosis* (ATCC 22019)	ATCC
*Nakaseomyces glabratus* resistant to fluconazole	60
*Saccharomyces cerevisiae* BY4742	Euroscarf

### Antifungal susceptibility

The MIC was determined by conducting broth microdilution assays adapted from the CLSI standard method M27-A3 (24). Serial dilutions of compound 5 and compound 8 were prepared in DMSO, resulting in a final concentration ranging from 2,500 μM to 2.4 μM. Growth in RPMI-1640 medium or SC medium pH 5.5, in the case of *S. cerevisiae*, which shows poor growth in RPMI, was recorded after 24 h of incubation at 37°C (*C. N. glabratus*) or 30°C (other *Candida* spp. and *S. cerevisiae*) by measuring OD_600_ using an Epoch Microplate Spectrophotometer and BioTek Gen5 Data Analysis Software. Growth without drug but with 2.5% DMSO (control condition) was used as the normalization condition, after background subtraction (RPMI-1640 medium). Three independent assays were performed. The MIC was defined as the concentration that inhibits at least 50% of cell growth.

### Quantification of intracellular nickel levels

*N. glabratus* cells were grown to the exponential phase and left untreated or treated overnight with the indicated concentrations of compound 5 or compound 8, 52.2 μM of fluconazole, or the combination of fluconazole and compound 5, overnight. After treatment, cells were harvested and washed with 10 mM EDTA and metal-free water. Total nickel intracellular contents were measured by ICP-AES at REQUIMTE LAQV, Universidade Nova de Lisboa, Caparica, Portugal, as previously detailed (28). Data were normalized against OD_600_. All assays were performed with four biological replicates.

### *In vivo* fungal burden

The *G. mellonella* caterpillar infection model was used for *in vivo* studies, following the assay described in reference 61 with minor modifications. *G. mellonella* larvae were reared on a diet of pollen grains and beeswax, maintained at 25°C in the dark, and used in a final stage of development (weight of approximately 200 mg). *N. glabratus* cultures were harvested by centrifugation and suspended in PBS. Each treatment and control group included six larvae per condition. Larvae were sequentially injected, via the hindmost left proleg sanitized with 70% (vol/vol) ethanol, with 5 μL of *N. glabratus* cells at a concentration of 1 × 10^9^ cells/mL, followed by a second injection with 5 μL of PBS (control), 5 μL of compound 5 at 5.6 mM (corresponding to 312.5 μM in the hemolymph, based on hemolymph volume of approximately 90 μL), or 5 μL of compound 8 at 5.6 mM. The injections were administered at 30-min intervals. The larvae were placed in Petri dishes and incubated in the dark at 37°C for 24 h. After incubation, the larvae were punctured in the abdomen with a sterile needle, and 10 μL of the hemolymph was collected and plated onto YPD agar plates supplemented with 100 μg/mL chloramphenicol for CFU counting.

### Mitochondrial potential

Cells were cultured at an initial concentration of 1.5 × 10^3^ cells/ml and left untreated or treated with 78.1 μM of compound 5 at 35°C. After 24 h, cells were washed and normalized to the lowest OD_600_ in 10 mM HEPES, pH 7.6, supplemented with 5% glucose. Cells were then resuspended in this buffer supplemented with 100 nM of DiOC6(3), incubated at room temperature for 15 min in the dark, and transferred to a NUNC microplate for fluorescence measurement. Confocal images were acquired using a Zeiss Axio Observer inverted widefield microscope equipped with an Apotome structured illumination module for optical sectioning. A 20 MP monochrome Axiocam 820 camera and a 63× Plan-Apochromat 1.4 NA DIC oil immersion objective were used. Image processing was performed using Fiji.

### Cytotoxicity

All experiments followed the ATCC Animal Cell Culture Guidelines (62). The animal cell line HeLa was cultured in Dulbecco’s modified Eagle’s medium (DMEM, Biowest) supplemented with 10% fetal bovine serum (Sigma-Aldrich) at 37°C in a 5% CO_2_ atmosphere. For cytotoxicity experiments, HeLa cells (5 × 10^3^ cells/well) were seeded in 96-well plates. After 24 h, different concentrations of compound 5 or compound 8 diluted in DMEM medium were added, and the plates were incubated for an additional 24 h. Cell viability was determined using 3-(4,5-dimethylthiazol-2-yl)−2,5-diphenyltetrazolium bromide (MTT) method (63). Culture medium was removed, MTT (0.5 mg/mL in DMEM) was added to each well and incubated for 2 h at 37°C. Formazan crystals were solubilized with DMSO for 15 min and quantified spectrophotometrically at 570 nm using the Epoch Microplate Spectrophotometer and BioTek Gen5 Data Analysis Software. HeLa cell viability was expressed as the percentage of treated cells relative to the control (cells cultured in DMEM with 0.5% DMSO). Assays were performed with four biological replicates.

### Checkerboard assays

Assays were performed according to the CLSI standard method M27-A3 for yeast, with minor modifications (24). Stock solutions of compound 5 and fluconazole were prepared in DMSO and ultrapure water, respectively. Twofold serial dilutions were prepared for each compound, resulting in a final concentration ranging from 0.6 to 625 µM for compound 5 and 6.5 to 417.9 µM for fluconazole. Growth in Synthetic Complete Medium pH 6.5 with 0.2% glucose was recorded after 24 h at 37°C by measuring OD_600_ using the Epoch Microplate Spectrophotometer and BioTek Gen5 Data Analysis Software and by spotting 5 μL of each well culture onto YPD agar plates. The type of interaction between compound 5 and fluconazole was determined by calculating the FIC index (∑FIC = FIC_5_ +FIC_Fluc_). For each combination, the FIC was calculated as follows: FIC_5_ = MIC_5+Fluc_/MIC_5_ and FIC_Fluc_ = MIC_Fluc+5_/MIC_Fluc_. A ∑FIC ≤ 0.5 indicates a synergistic effect.

### Biofilm assays

The effect of compound 5 alone or combined with fluconazole on *N. glabratus* biofilm was assessed as previously described (64) with minor modifications. Briefly, 100 µL of exponentially growing cells diluted to a concentration of 1 × 10^7^ cells/mL was added to each well of a 96-well plate and incubated for 90 min at 37°C to allow adherence. Non-adherent cells were aspirated, the wells were washed with PBS, and RPMI-1640, containing 78.1 μM of compound 5, 52.2 μM of fluconazole, or the combination of both, was added. The plate was incubated for an additional 24 h at 37°C, and the inhibitory effect of the compound was evaluated by staining the biofilms with 0.4% of crystal violet (65). The assay was performed with eight biological replicates.

### ROS measurements

Cells were grown to exponential phase and left untreated or treated with 78.1 μM of compound 5 or with 10 mM hydrogen peroxide for 2 h. Cultures were harvested, and the OD_600_ was normalized to the lowest value. Cells were resuspended in PBS containing 5 μg/mL of dihydrorhodamine 123 and incubated at room temperature in the dark for 2 h. After incubation, cells were washed three times with PBS and transferred to a black NUNC microplate for fluorescence measurement (Ex: 498 nm and Em: 539 nm) using the BioTek Plate Reader Neo2 Multimode Reader. The assay was performed with four biological replicates.

## Supplementary Material

Reviewer comments

## Data Availability

All data generated or analyzed during this study are included in this published article and its supplemental material.

## SUPPLEMENTAL MATERIAL

Supplemental figures spectrum.00759-26-s0001.pdf Fig. S1 to S5.

OPEN PEER REVIEW reviewer-comments.pdf An accounting of the reviewer comments and feedback.

ASM does not own the copyrights to Supplemental Material that may be linked to, or accessed through, an article. The authors have granted ASM a non-exclusive, world-wide license to publish the Supplemental Material files. Please contact the corresponding author directly for reuse.
