# Supplementary material for: A nickel N-heterocyclic biscarbene complex derived from caffeine enhances fluconazole efficacy against Nakaseomyces glabratus
Source: Microbiol Spectr. 2026 Sep 2;14(10):e00759-26. doi: 10.1128/spectrum.00759-26 (PMC13688839; doi:10.1128/spectrum.00759-26)
Supplement: Supplementary file 1 [file reviewer-comments.pdf]

ASM | Microbiology Spectrum

A Nickel N-Heterocyclic Biscarbene Complex Derived from Caffeine Enhances Fluconazole Efficacy against *Nakaseomyces glabratus*

Cláudia Malta-Luís, Giulia Romeo, Giulia Francescato, Carolina V. Mariano, Dalila Mil-Homens, Ana Petronilho, and Catarina Pimentel

Corresponding Author(s): Catarina Pimentel, Universidade Nova de Lisboa Instituto de Tecnologia Quimica e Biologica

Review Timeline:

Submission Date:	March 4, 2026
Editorial Decision:	May 10, 2026
Revision Received:	July 27, 2026
Accepted:	August 11, 2026

Editor: Justyna Karkowska-Kuleta

Reviewer(s): The reviewers have opted to remain anonymous.

Transaction Report:

(Note: With the exception of the correction of typographical or spelling errors that could be a source of ambiguity, letters and reports are not edited. The original formatting of letters and referee reports may not be reflected in this compilation.)

DOI: https://doi.org/10.1128/spectrum.00759-26

Re: Spectrum00759-26 (**A Nickel N-Heterocyclic Biscarbene Complex Derived from Caffeine Enhances Fluconazole Efficacy against *Candida glabrata***)

Dear Dr. Catarina Pimentel:

Thank you for the privilege of reviewing your work. Below you will find my comments, instructions from the Spectrum editorial office, and the reviewer comments.

The reviewers considered the manuscript an interesting presentation of valuable results; however, they also acknowledged that more mechanistic insight is required to substantiate the proposed claims. In addition, calculation of the FICI values for the tested compound and the antifungal drug is necessary, along with the implementation of several other revisions.

Please return the manuscript within 60 days; if you cannot complete the modification within this time period, please contact me. If you do not wish to modify the manuscript and prefer to submit it to another journal, notify me immediately so that the manuscript may be formally withdrawn from consideration by Spectrum.

Revision Guidelines
To submit your modified manuscript, log into the submission site at https://spectrum.msubmit.net/cgi-bin/main.plex. Go to Author Tasks and click the appropriate manuscript title to begin. The information you entered when you first submitted the paper will be displayed; update this as necessary. Note the following requirements:

• Upload point-by-point responses to the issues raised by the reviewers in a file named "Response to Reviewers," NOT in your cover letter.
• Upload a compare copy of the manuscript (without figures) as a "Marked-Up Manuscript" file.
• Upload a clean .DOC/.DOCX version of the revised manuscript and remove the previous version.
• Each figure must be uploaded as a separate, editable, high-resolution file (TIFF or EPS preferred), and any multipanel figures must be assembled into one file.
• Any supplemental material intended for posting by ASM should be uploaded with their legends separate from the main manuscript. You can combine all supplemental material into one file (preferred) or split it into a maximum of 10 files with all associated legends included.

For complete guidelines on revision requirements, see our Submission and Review Process webpage. Submission of a paper that does not conform to guidelines may delay acceptance of your manuscript.

Data availability: ASM policy requires that data be available to the public upon online posting of the article, so please verify all links to sequence records, if present, and make sure that each number retrieves the full record of the data. If a new accession number is not linked or a link is broken, provide Spectrum production staff with the correct URL for the record. If the accession numbers for new data are not publicly accessible before the expected online posting of the article, publication may be delayed; please contact production staff (Spectrum@asmusa.org) immediately with the expected release date.

Publication Fees: For information on publication fees and which article types are subject to charges, visit our website. If your manuscript is accepted for publication and any fees apply, you will be contacted separately about payment during the production process; please follow the instructions in that e-mail. Arrangements for payment must be made before your article is published.

ASM Membership: Corresponding authors may join or renew ASM membership to obtain discounts on publication fees. Need to upgrade your membership level? Please contact Customer Service at Service@asmusa.org.

The ASM Journals program strives for constant improvement in our submission and publication process. Please tell us how we can improve your experience by taking this quick Author Survey.

Thank you for submitting your paper to Spectrum.

Sincerely,
Justyna Karkowska-Kuleta
Editor
Microbiology Spectrum

Reviewer #1 (Comments for the Author):

In a previous study, the authors identified a nickel N-heterocyclic biscarbene complex derived from caffeine (compound 5). This compound exhibited selective activity against C. glabrata and low in vitro cytotoxicity.

In the present study, Malta-Luís et al. synthesized a benzimidazole-based N-heterocyclic biscarbene (compound 8), which showed greater in vitro but not improved in vivo efficacy compared with compound 5. The results further indicate that the addition of an extra carbene moiety alone is insufficient to reduce cytotoxicity. Based on these findings, the authors proceeded with compound 5 for further biological characterization. However, the manuscript is not yet ready for publication.

The study addresses a topic of relevance and general interest to the journal's readership. Nevertheless, several issues should be addressed to improve the quality of the manuscript prior to publication

Major Comments:
1. Mechanistic claims should be moderated. The authors propose mitochondrial involvement, possible mtDNA interaction, and fluconazole-enhanced uptake; however, the evidence remains suggestive rather than conclusive. While ROS involvement is ruled out, no direct mitochondrial assays (e.g. membrane potential, respiration, mtDNA quantification) are provided. The mtDNA-biding hypothesis is largely speculative and based on caffeine-related literature.

2. Synergy analysis lack quantitative rigor. Fractional Inhibitory Concentration Index (FICI) values should be calculated and reported.

3. Clarify the comparison between compounds 5 and 8. Although compound 5 performs better in vivo and is less toxic, compound 8 shows higher in vitro potency. The proposed explanation based on lipophilicity remains speculative, as no pharmacokinetic or distribution data are presented. A more appropriate statement would be: "In vitro potency does not necessarily translate into in vivo efficacy, possibly due to physicochemical constraints such as lipophilicity.

4. The petite mutant paradox needs clarification.

5. Translational relevance is overstated. Claims such as "promising therapeutic lead" (line 56) and "potential use in combination therapy" (line 45) are too strong given the absence of a mammalian infection model, pharmacokinetic data, and comprehensive toxicity evaluation beyond HeLa cells. These should be softened, for example: "represents a promising candidate for further preclinical evaluation"

Minor Comments
Line 12: "Address correspondence"

Line 76, 82, 146, 170, 173, 201, 276, 340: use "Candida spp." consistently.

Ensure consistency in terminology: use full names on first mention (eg. Candida glabrata, Candida albicans, Galleria mellonella), followed by abbreviations (C. glabrata, C. albicans, G. mellonella) thereafter.

Since only one newly synthesized compound (compound 8) is reported, consider assigning it a more descriptive name to avoid the impression that multiple compounds were synthesized.

If the nomenclature "compound 5" and "compound 8" is retained, ensure consistency:
Line 140: "(5)" should be written as "(compound 5)", to avoid confusion with references.
Apply this correction throughout the manuscript:
Lines 35, 44, 145, 150, 161,163,164,166, 172, 174, 181, 184, 192, 197, 199, 204, 210, 263, 265, 277, 278, 286, 294, 296, 304, 356, 369, 370, 422: compound 5
Lines 145, 263, 266, 267, 356, 369: compound 8

Line 263: revise to "Although compound 8 showed (...) than compound 5

Line 298: use either "to this azole" or "to fluconazole"

Line 358: correct typo

Lines 762, 769, 788, 793, 813, 819, and figure legends (Figures 2, 3, 5): standardize to "Tukey's HSD"

Methods: Standardize reporting of replicates (currently inconsistent)

Minor inconsistencies in references: check journal abbreviations and DOI formatting

Reviewer #2 (Comments for the Author):

The article is interesting but descriptive, with little or no mechanistic insights. Candida glabrata is one of the common human fungal pathogen and fluconazole resistance is a huge problem while treating C. glabrata infections. The authors discussed a nickel-based caffeine-derived compound, which enhances fluconazole efficacy. Here are my comments:

1) The authors observed petite phenotypes but did not do any assays linked to mitochondrial functions. This needs clarification.

2)While describing synergism, the authors did not analyze FICI values between fluconazole and compound 5. This needs to be analyzed to make the manuscript stronger.

3)Fig 5B: The authors did not analyze the p-values between compound 5 and compound 5+fluconazole. Compound 5 alone may have a similar effect on biofilm biomass with or without fluconazole. This needs to be discussed.

4)Fig 2 should reflect the sample size in the figure legend for better clarity. Were all 30 larvae punctured and plated?

5)Cytotoxicity for compounds 5 and 8 was performed in HeLa cells. However, since the authors proposed the use of compound 5 along with fluconazole, the cytotoxicity in HeLa should have been analyzed in the presence of fluconazole. Please explain.

6) Fig 2A : is there any statistical difference in CFU/mL between compounds 5 and 8? Please clarify since the authors claimed that there are reduced activities of compound 8 in vivo. Please clarify.

Reviewer #3 (Comments for the Author):

This is a well-written manuscript with a clear and concise presentation. The study is well aligned with an important public health concern, namely antifungal resistance. The introduction is particularly strong and provides good context for the study. Below are a few comments for the authors' consideration.

1. Concerning the title: The nomenclature of the yeast should be updated to reflect current taxonomy. This is particularly important in a basic science context, where accurate and up-to-date organism identification is essential. The previous nomenclature may be retained in the manuscript keywords to aid discoverability.

2. In lines 35 and 44, compound 5 is referred to only as "5". It should be properly referenced as "Compound 5" as introduced for clarity.

3. The term "petite" should not be italicized, as it refers to a phenotypic variant rather than a taxonomic designation.

4. The authors report synergistic activity between Compound 5 and fluconazole in C. glabrata. While the selective activity of Compound 5 may justify focusing on this species, it would be helpful for the authors to clarify why combination studies were not extended to other Candida spp. or to Saccharomyces cerevisiae, particularly given that synergistic effects can occur even in yeasts with limited susceptibility to the compound alone (example is seen in Cowen, L.E., Singh, S.D., Köhler, J.R., Collins, C., Zaas, A.K., Schell, W.A., Aziz, H., Mylonakis, E., Perfect, J.R., Whitesell, L. and Lindquist, S., 2009. Harnessing Hsp90 function as a powerful, broadly effective therapeutic strategy for fungal infectious disease. Proceedings of the National Academy of Sciences, 106(8), pp.2818-2823.)

5. The synergistic effect of Compound 5 was evaluated only in combination with fluconazole. It would be helpful for the authors to comment on whether similar interactions might be expected with other azoles or antifungal agents from different classes. Expanding or discussing this aspect would strengthen the broader relevance of the findings.

This is a well-written manuscript with a clear and concise presentation. The introduction is particularly strong and provides good context for the study. Below are a few comments for the authors' consideration.

1. Concerning the title: The nomenclature of the yeast should be updated to reflect current taxonomy. This is particularly important in a basic science context, where accurate and up-to-date organism identification is essential. The previous nomenclature may be retained in the manuscript keywords to aid discoverability.

2. In lines 35 and 44, compound 5 is referred to only as "5". It should be properly referenced as "Compound 5" as introduced for clarity.

3. The term "petite" should not be italicized, as it refers to a phenotypic variant rather than a taxonomic designation.

4. The authors report synergistic activity between Compound 5 and fluconazole in *C. glabrata*. While the selective activity of Compound 5 may justify focusing on this species, it would be helpful for the authors to clarify why combination studies were not extended to other *Candida* spp. or to *Saccharomyces cerevisiae*, particularly given that synergistic effects can occur even in yeasts with limited susceptibility to the compound alone (example is seen in Cowen, L.E., Singh, S.D., Köhler, J.R., Collins, C., Zaas, A.K., Schell, W.A., Aziz, H., Mylonakis, E., Perfect, J.R., Whitesell, L. and Lindquist, S., 2009. Harnessing Hsp90 function as a powerful, broadly effective therapeutic strategy for fungal infectious disease. Proceedings of the National Academy of Sciences, 106(8), pp.2818-2823.)

5. The synergistic effect of Compound 5 was evaluated only in combination with fluconazole. It would be helpful for the authors to comment on whether similar interactions might be expected with other azoles or antifungal agents from different classes. Expanding or discussing this aspect would strengthen the broader relevance of the findings.

The authors thank the reviewers for their insightful suggestions and helpful comments, which have contributed to improving the manuscript.

Below, we address all of the reviewers' concerns in detail. A revised version of the manuscript is also provided, with modifications marked using track changes to reflect all suggestions and comments.

____

Reviewer 1

1. "Mechanistic claims should be moderated. The authors propose mitochondrial involvement, possible mtDNA interaction, and fluconazole-enhanced uptake; however, the evidence remains suggestive rather than conclusive. While ROS involvement is ruled out, no direct mitochondrial assays (e.g. membrane potential, respiration, mtDNA quantification) are provided. The mtDNA-biding hypothesis is largely speculative and based on caffeine-related literature."

In the revised version, the authors have refrained from making mechanistic claims, as suggested by the Reviewer. The authors also agree that the hypothesis of mitochondrial dysfunction required stronger experimental support than the inability to grow on non-fermentable carbon sources and the available literature alone. Therefore, we performed mitochondrial membrane potential assays in the presence and absence of compound 5. Supporting the authors' hypothesis, the revised manuscript demonstrates that compound 5 induces mitochondrial depolarization. Accordingly, two new panels have been added to Figure 6 to present these data, and the Results section has been expanded as follows:

"In addition, we assessed the mitochondrial membrane potential of yeast cells left untreated or treated with compound 5 using the fluorescent probe DiOC6(3) (Fig. 6C,D). Treatment with compound 5 led to a decrease in DiOC6(3) fluorescence, indicating a loss of mitochondrial membrane potential and suggesting mitochondrial dysfunction. This finding is consistent with the ability of compound 5 to induce the formation of petite mutants, further supporting an adverse effect on mitochondrial function."

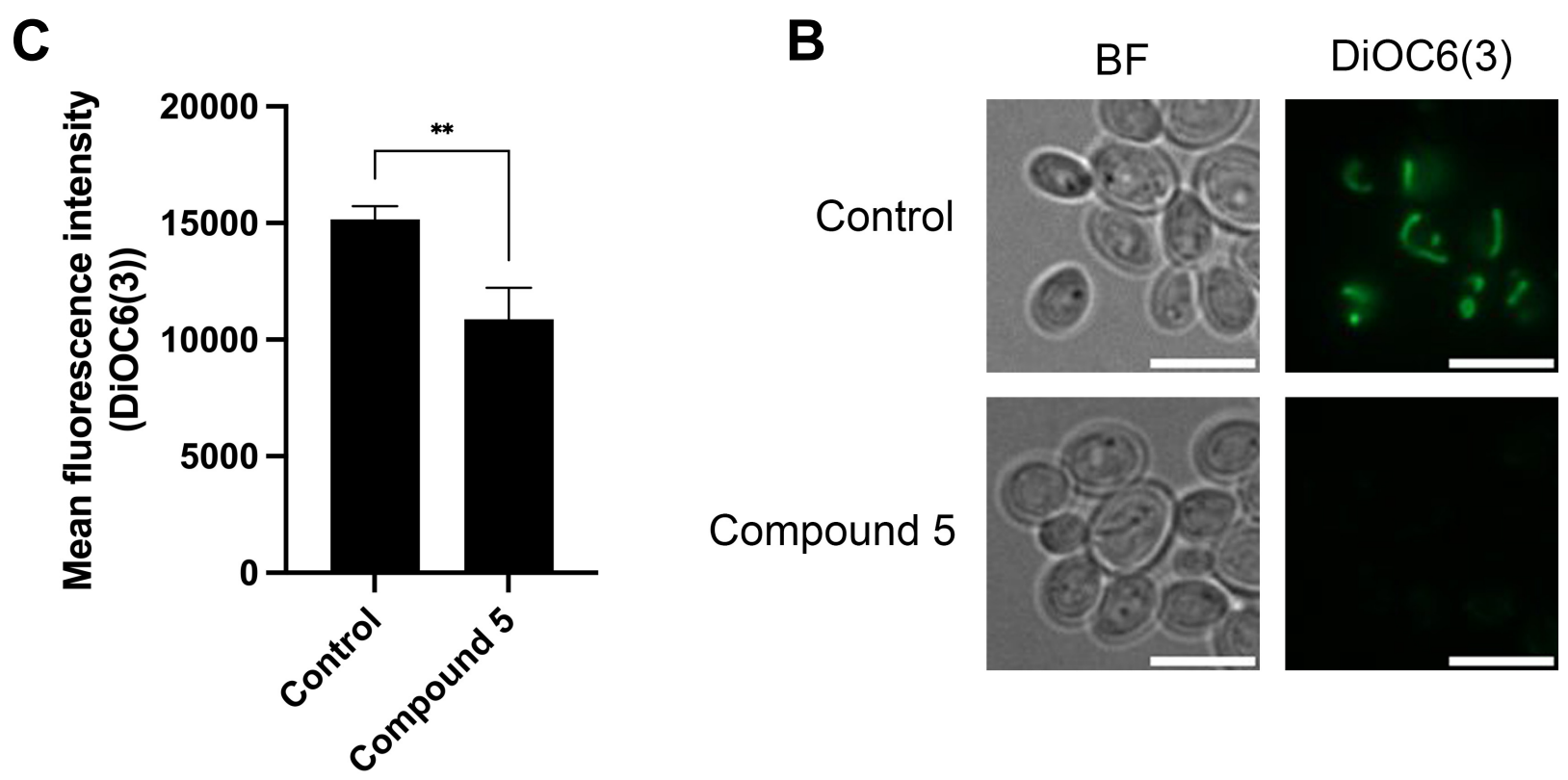

Figure 6 (new panels C and D). Mitochondrial membrane potential is affected by compound 5. (C) Changes in mitochondrial membrane potential in *C. glabrata* cells, untreated (Control) or treated with 78.1 µM of compound 5, quantified using the fluorescent dye DiOC6(3). Values are the mean of three biological replicates. Statistical significance was assessed using Student's T-test (**p < 0.01). (D) Confocal microscopy images of *C. glabrata* cells either left untreated (Control) or treated with 78.1 µM of compound 5, stained with DiOC6(3) to visualize mitochondria. The scale bar represents 5 µm.

2. "Synergy analysis lack quantitative rigor. Fractional Inhibitory Concentration Index (FICI) values should be calculated and reported."

The Reviewer is correct. We have repeated all checkerboard experiments and determined the Fractional Inhibitory Concentration Index (ΣFIC) as suggested. We have added a new table (Table 4, below) in which the ΣFIC values are presented and the nature of the interaction (synergistic) is clearly stated.

Table 4. *Antifungal susceptibility testing and drug interaction evaluation in fluconazole-susceptible and fluconazole-resistant C. glabrata strains.*

	Standard MIC (µM)		Combination MIC (µM)		ΣFIC	Interaction
	5	Fluconazole	5	Fluconazole		
C. glabrata ATCC2001	78.1	26.1	39	13.1	0.5	Synergy
C. glabrata resistant to fluconazole	78.1	209	39	104.5	0.5	Synergy
Compound 5-induced petite *C. glabrata* mutant	78.1	1671.6	39	835.8	0.031	Synergy

3. "Clarify the comparison between compounds 5 and 8. Although compound 5 performs better in vivo and is less toxic, compound 8 shows higher in vitro potency. The proposed explanation based on lipophilicity remains speculative, as no pharmacokinetic or distribution data are presented. A more appropriate statement would be: "In vitro potency does not necessarily translate into in vivo efficacy, possibly due to physicochemical constraints such as lipophilicity".

We have revised this section of the Discussion in accordance with the Reviewer's suggestion to make it less speculative. In addition, we have removed Figure S3, as it is no longer relevant in the revised context.

4. "The petite mutant paradox needs clarification."

We did not fully understand the Reviewer's comment. The ability of compound 5 to induce petite mutants was unexpected because, in *C. glabrata*, petite mutants are less susceptible to fluconazole. This reduced susceptibility is thought to result from mitochondrial dysfunction, which increases the expression of drug efflux pumps that remove fluconazole from the cell. However, our results suggest that compound 5, while inducing the formation of petite mutants, also exerts additional effects that overcome the potential reduced susceptibility. This interpretation is supported by two observations: (i) the synergistic interaction with fluconazole is maintained in petite mutants, and (ii) co-treatment with fluconazole results in a markedly lower proportion of petite mutants than treatment with compound 5 alone. Together, these findings suggest that, although compound 5 induces petite mutants, its overall effect enhances the activity of fluconazole.

5. "Translational relevance is overstated. Claims such as "promising therapeutic lead" (line 56) and "potential use in combination therapy" (line 45) are too strong given the absence of a mammalian infection model, pharmacokinetic data, and comprehensive toxicity evaluation beyond HeLa cells. These should be softened, for example: "represents a promising candidate for further preclinical evaluation"

The revised manuscript has been modified accordingly.

Minor Comments

"Line 12: "Address correspondence"

Corrected

"Line 76, 82, 146, 170, 173, 201, 276, 340: use "Candida spp." consistently"

Corrected.

"Ensure consistency in terminology: use full names on first mention (eg. Candida glabrata, Candida albicans, Galleria mellonella), followed by abbreviations (C. glabrata, C. albicans, G. mellonella) thereafter."

Done.

“Since only one newly synthesized compound (compound 8) is reported, consider assigning it a more descriptive name to avoid the impression that multiple compounds were synthesized.”
For consistency with our ongoing chemical follow-up studies, the authors have chosen to retain this nomenclature.

“If the nomenclature "compound 5" and "compound 8" is retained, ensure consistency: Line 140: "(5)" should be written as "(compound 5)", to avoid confusion with references.”
Corrected, accordingly.

“Line 263: revise to "Although compound 8 showed (...) than compound 5”
Corrected.

“Line 298: use either "to this azole" or "to fluconazole"
Corrected.

“Line 358: correct typo”
Corrected.

“Lines 762, 769, 788, 793, 813, 819, and figure legends (Figures 2, 3, 5): standardize to "Tukey's HSD"
Corrected.

Corrected.

Minor inconsistencies in references: check journal abbreviations and DOI formatting –
Thank you for spotting this. We have downloaded the EndNote style file provided by the journal and reformatted all references accordingly.

Reviewer 2

1) “The authors observed petite phenotypes but did not do any assays linked to mitochondrial functions. This needs clarification.”
We agree with this criticism, which was also raised by Reviewer 1, and have revised the manuscript accordingly by including new experimental data (please refer to our response to Reviewer 1, point 1). These results are presented in the new panels B and C of Figure 6.

2) ”While describing synergism, the authors did not analyze FICI values between fluconazole and compound 5. This needs to be analyzed to make the manuscript stronger.”

This issue was also raised by Reviewer 1, and, in accordance with the suggestions of both reviewers, we determined the FICI (please refer to our response to Reviewer 1, point 2). The results are presented in the new Table 4.

3) "Fig 5B: The authors did not analyze the p-values between compound 5 and compound 5+fluconazole. Compound 5 alone may have a similar effect on biofilm biomass with or without fluconazole. This needs to be discussed"

This was indeed missing from the previous analysis. The difference in biofilm formation between compound 5 alone and the combination of fluconazole and compound 5 is statistically significant. In the revised manuscript, this statistical significance is now clearly indicated in the figure.

4)"Fig 2 should reflect the sample size in the figure legend for better clarity. Were all 30 larvae punctured and plated? "

We have modified the figure to indicate the number of larvae analyzed. During this revision, we also identified an inconsistency. Although 10 larvae were included for each condition, due to the coagulation of hemolymph provoked by the fungal burden, sufficient hemolymph could be collected from only 6 larvae per condition. This occurred coincidentally across all experimental groups! We have corrected this information in both the figure and the Materials and Methods section.

6) Fig 2A : is there any statistical difference in CFU/mL between compounds 5 and 8? Please clarify since the authors claimed that there are reduced activities of compound 8 in vivo. Please clarify.

When the effects of compounds 5 and 8 were analyzed in isolation using Student's *t*-test, the difference between the two compounds was statistically significant (p = 0.0481), Figure R2 below. However, when the data were analyzed using a one-way ANOVA, which is the appropriate approach for comparing more than two experimental groups simultaneously, this pairwise difference was no longer statistically significant (p = 0.126). Notably, the difference between the control group and compound 8 was also not statistically significant in the ANOVA analysis (new revised Fig.2 with all analyzes). Therefore, we have retained the statement that compound 8 is less active *in vivo*, while acknowledging that the difference between compounds 5 and 8 does not reach statistical significance under the more appropriate ANOVA-based analysis.

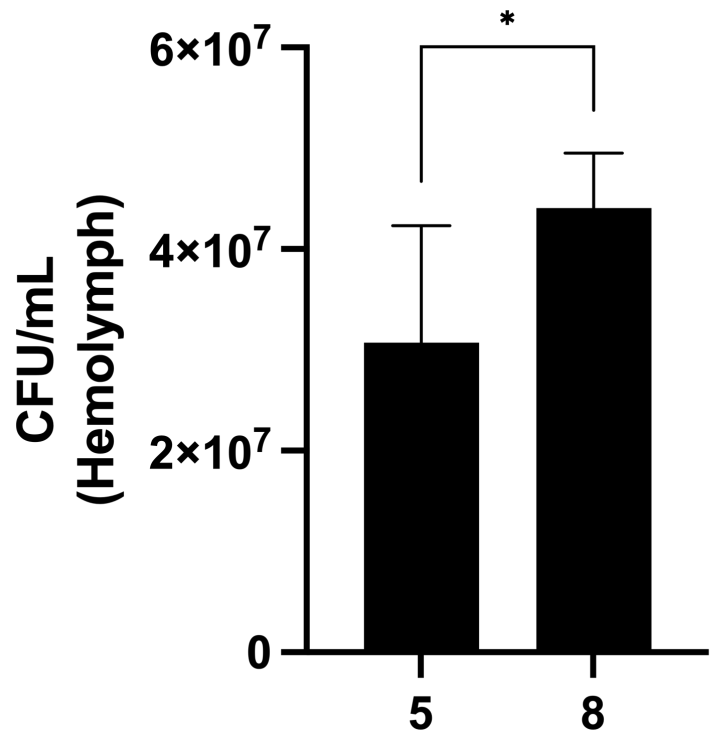

Figure R2. Fungal burden in the hemolymph of infected *G. mellonella* treated with 312.5 µM of compound **5** (**5**) or 312.5 µM of compound **8** (**8**). Statistical significance was assessed using Student's T-test (*p≤0.05).

5) "Cytotoxicity for compounds 5 and 8 was performed in HeLa cells. However, since the authors proposed the use of compound 5 along with fluconazole, the cytotoxicity in HeLa should have been analyzed in the presence of fluconazole. Please explain."

This has been addressed in the revised version of the manuscript. No toxicity was observed for the combination of fluconazole and compound 5 over a wide range of concentrations, as shown in the new Figure S4 (below).

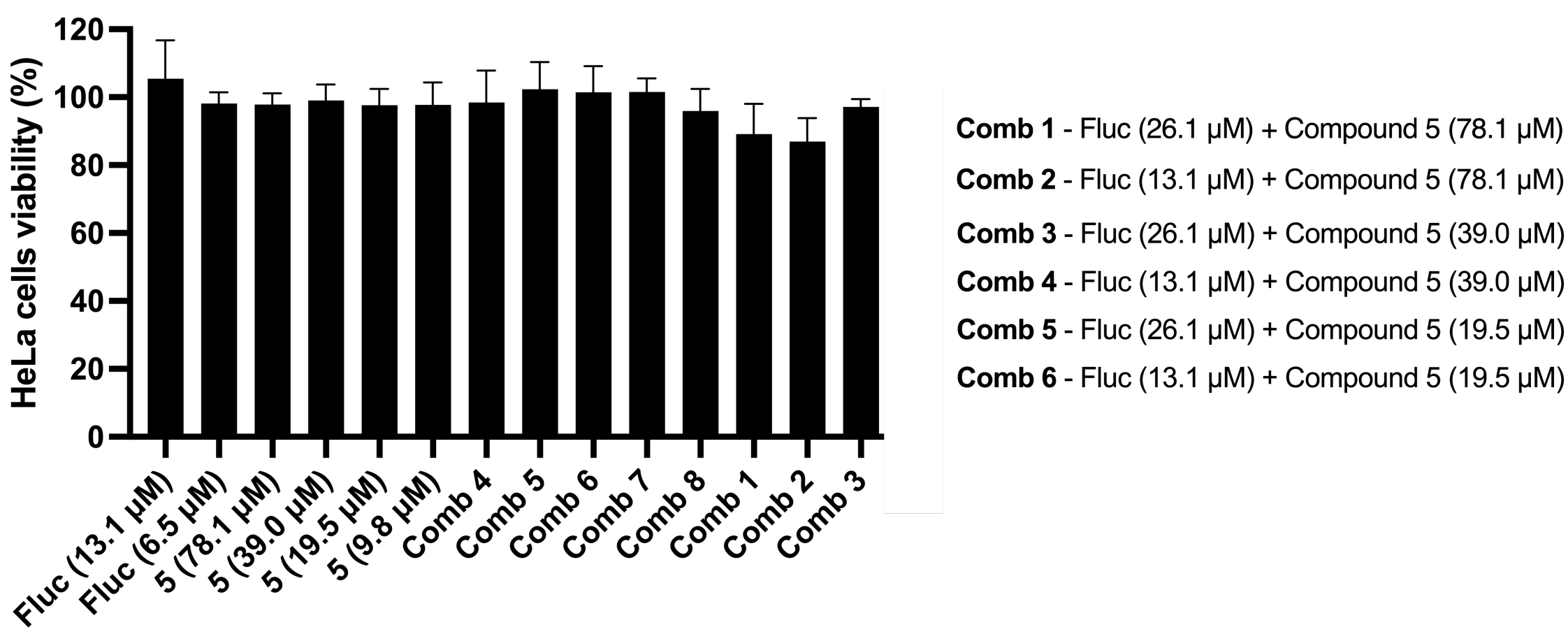

Figure S4. The combination of compound 5 with fluconazole is not cytotoxic against mammalian cells. Cellular viability after treatment with the indicated concentrations of fluconazole (Fluc), compound 5 (5) or their combination (Comb) was evaluated using the MTT assay and the values are expressed as the percentage of viability relative to the untreated control.

Reviewer 3

1. "Concerning the title: The nomenclature of the yeast should be updated to reflect current taxonomy. This is particularly important in a basic science context, where

accurate and up-to-date organism identification is essential. The previous nomenclature may be retained in the manuscript keywords to aid discoverability."

Following the Reviewer's suggestion, we have replaced *Candida glabrata* with *Nakaseomyces glabratus* throughout the manuscript.

2. "In lines 35 and 44, compound 5 is referred to only as "5". It should be properly referenced as "Compound 5" as introduced for clarity."

This has been corrected accordingly.

3. "The term "petite" should not be italicized, as it refers to a phenotypic variant rather than a taxonomic designation."

Thank you. This has been corrected throughout the manuscript.

4. "The authors report synergistic activity between Compound 5 and fluconazole in C. glabrata. While the selective activity of Compound 5 may justify focusing on this species, it would be helpful for the authors to clarify why combination studies were not extended to other Candida spp. or to Saccharomyces cerevisiae, particularly given that synergistic effects can occur even in yeasts with limited susceptibility to the compound alone (example is seen in (…)) "

We followed the Reviewer's suggestion and tested the synergistic interaction in *S. cerevisiae*. No synergistic effect was observed (Figure R3, below). Due to limited compound availability and the high MIC values for the other *Candida* species, we were unable to extend this analysis to those organisms.

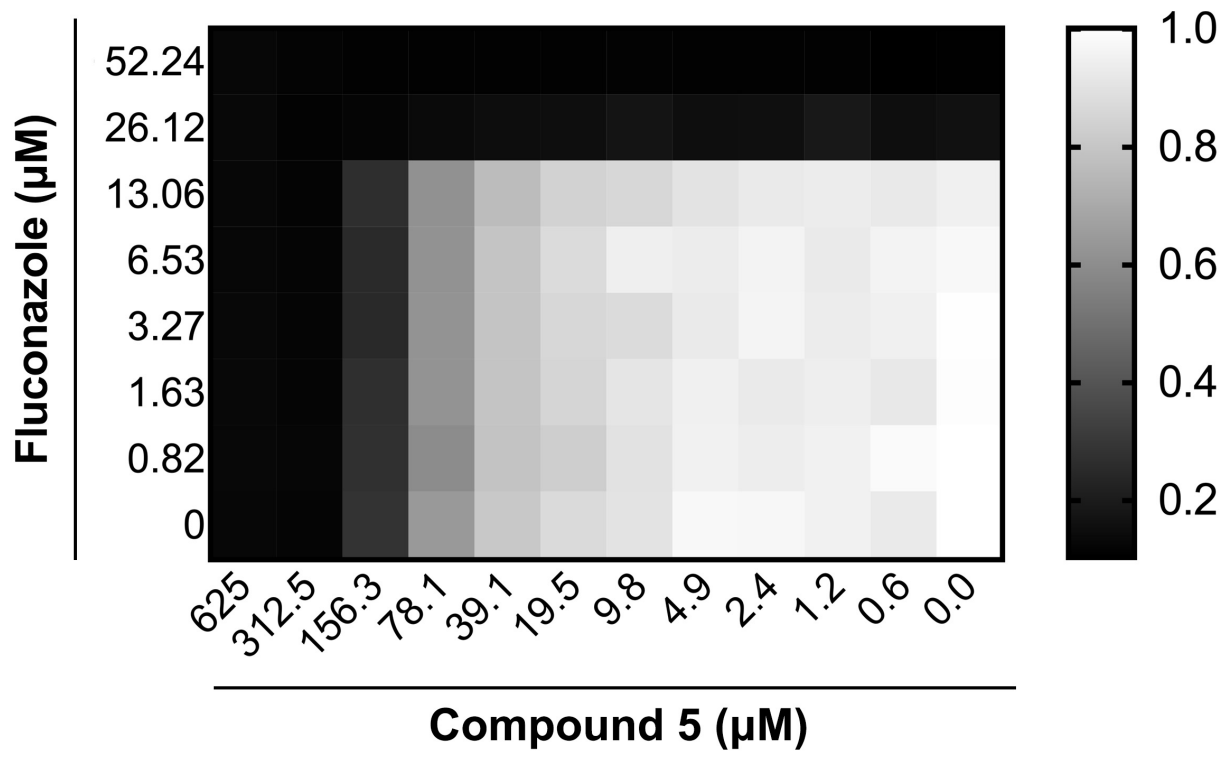

Figure R3. No interaction between compound 5 and fluconazole was observed in *Saccharomyces cerevisiae.*

5. "The synergistic effect of Compound 5 was evaluated only in combination with fluconazole. It would be helpful for the authors to comment on whether similar interactions might be expected with other azoles or antifungal agents from different

classes. Expanding or discussing this aspect would strengthen the broader relevance of the findings. "

The authors appreciate the Reviewer's comment. Checkerboard assays were performed in *C. glabrata* using representatives of other antifungal classes, including amphotericin B (polyenes), caspofungin (echinocandins), and another member of the azole class, the triazole voriconazole. The synergistic effect appeared to be fluconazole-specific, as it was not observed with the other antifungal agents tested. This information has been incorporated into the revised manuscript and is presented in the new Figure S3 (below).

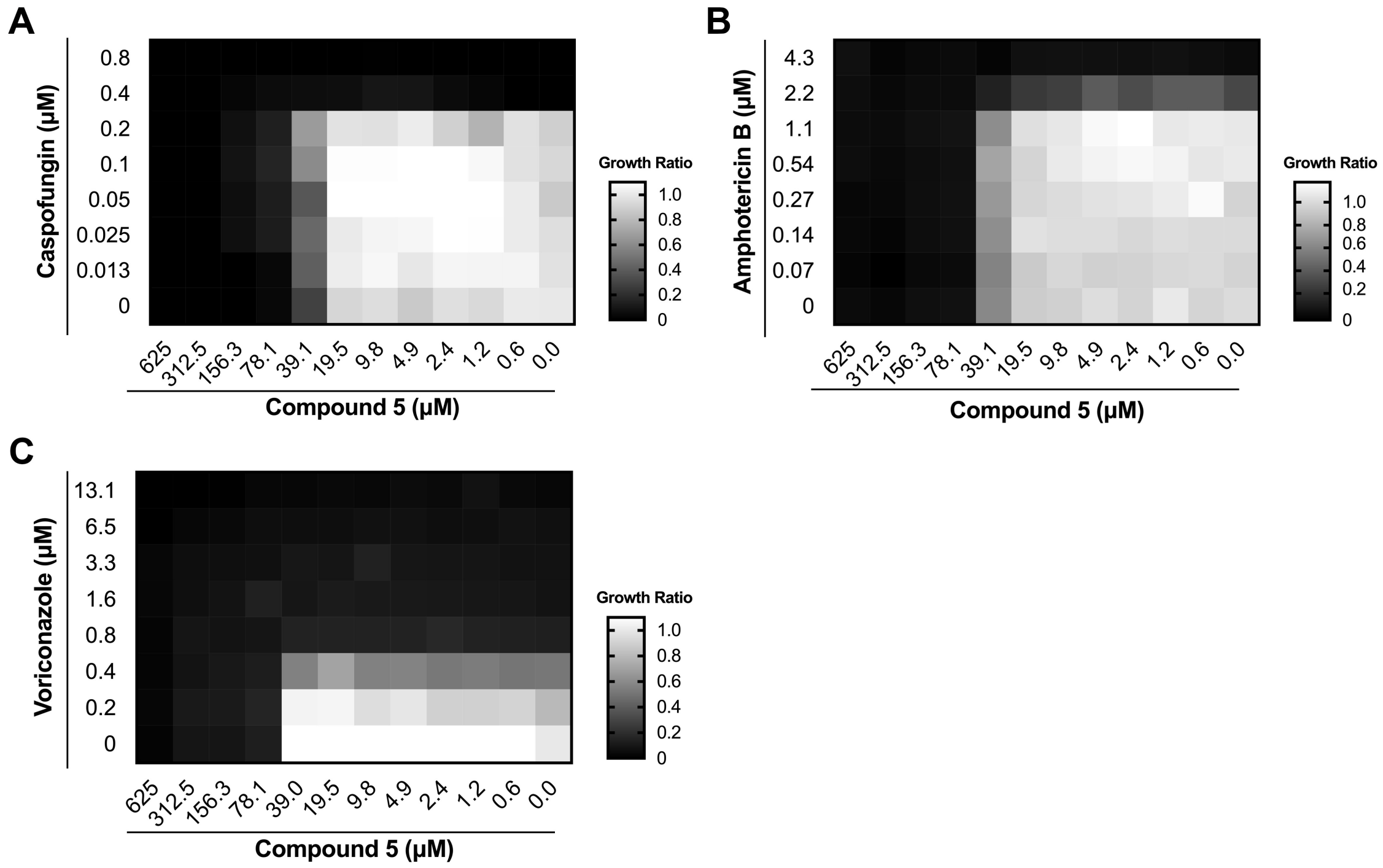

Figure S3. Compound 5 does not synergistically interact with other antifungal drugs. Checkerboard assays were performed to evaluate the growth of *C. glabrata* in medium supplemented with compound 5 combined with caspofungin (A), amphotericin B (B) or voriconazole (C). After 24 h, OD_{600} was measured, values were normalized to untreated controls.

Re: Spectrum00759-26R1 (**A Nickel N-Heterocyclic Biscarbene Complex Derived from Caffeine Enhances Fluconazole Efficacy against *Nakaseomyces glabratus***)

Dear Dr. Catarina Pimentel:

Your manuscript has been accepted, and I am forwarding it to the ASM production staff for publication. Your paper will first be checked to make sure all elements meet the technical requirements. ASM staff will contact you if anything needs to be revised before copyediting and production can begin. Otherwise, you will be notified when your proofs are ready to be viewed.

Data Availability: ASM policy requires that data be available to the public upon online posting of the article, so please verify all links to sequence records, if present, and make sure that each number retrieves the full record of the data. If a new accession number is not linked or a link is broken, provide production staff with the correct URL for the record. If the accession numbers for new data are not publicly accessible before the expected online posting of the article, publication may be delayed; please contact ASM production staff immediately with the expected release date.

Publication Fees: For information on publication fees and which article types have charges, please visit ourwebsite. We have partnered with Copyright Clearance Center (CCC) to collect author charges. If fees apply to your paper, you will receive a message from no-reply@copyright.com with further instructions. For questions related to paying charges through RightsLink, please contact CCC at ASM_Support@copyright.com or toll free at +1-877-622-5543. CCC makes every attempt to respond to all emails within 24 hours.

ASM Membership: Corresponding authors may join or renew ASM membership to obtain discounts on publication fees. Need to upgrade your membership level? Please contact Customer Service at Service@asmusa.org.

Embargo Policy: A press release may be issued as soon as the manuscript is posted on theSpectrum Latest Articles webpage. The corresponding author will receive an email with the subject line "ASM Journals Author Services Notification" when the article is available online.

Cover Image Submissions: If you would like to submit a potential Cover Image, please email a file and a short legend to spectrum@asmusa.org. Please note that we can only consider images that (i) the authors created or own and (ii) have not been previously published. By submitting, you agree that the image can be used under the same terms as the published article. Image File requirements: TIF/EPS, 7.5 inches wide by 8.25 inches tall (at least 2,250 pixels wide by 2,475 pixels tall), minimum 300 dpi resolution (600 dpi preferred), RGB, and no figure elements, e.g., arrows or panel labels. The legend should be a short description of the image, 1-2 sentences recommended. Please use the ASM Journals Cover Image Sizing Tool (https://journals.asm.org/pb-assets/html/asm-cover-sizing-tool.html) to ensure that your proposed cover image meets our size requirements.

The ASM Journals program strives for constant improvement in our submission and publication process. Please tell us how we can improve your experience by taking this quick Author Survey.

Thank you for submitting your paper to Spectrum.

Sincerely,
Justyna Karkowska-Kuleta
Editor
Microbiology Spectrum

Reviewer #1 (Comments for the Author):

All comments and suggestions were addressed.

Reviewer #2 (Comments for the Author):

The authors have answered my comments satisfactorily, and the manuscript has improved substantially.
