# Supplementary material for: A nickel N-heterocyclic biscarbene complex derived from caffeine enhances fluconazole efficacy against Nakaseomyces glabratus
Source: Microbiol Spectr. 2026 Sep 2;14(10):e00759-26. doi: 10.1128/spectrum.00759-26 (PMC13688839; doi:10.1128/spectrum.00759-26)
Supplement: Supplementary file 2 [file spectrum.00759-26-s0001.pdf]

Supplementary Figures

Title: A Nickel N-Heterocyclic Biscarbene Complex Derived from Caffeine Enhances Fluconazole Efficacy against *Nakaseomyces glabratus*

[1]Cláudia Malta-Luís, [1]Giulia Romeo, [1]Giulia Francescato, [1]Carolina Mariano, [1]Dalila Mil-Homens [1*]Ana Petronilho and [1*]Catarina Pimentel

[1] Instituto de Tecnologia Química e Biológica António Xavier, Universidade Nova de Lisboa, Av. República, 2780-157 Oeiras, Portugal.

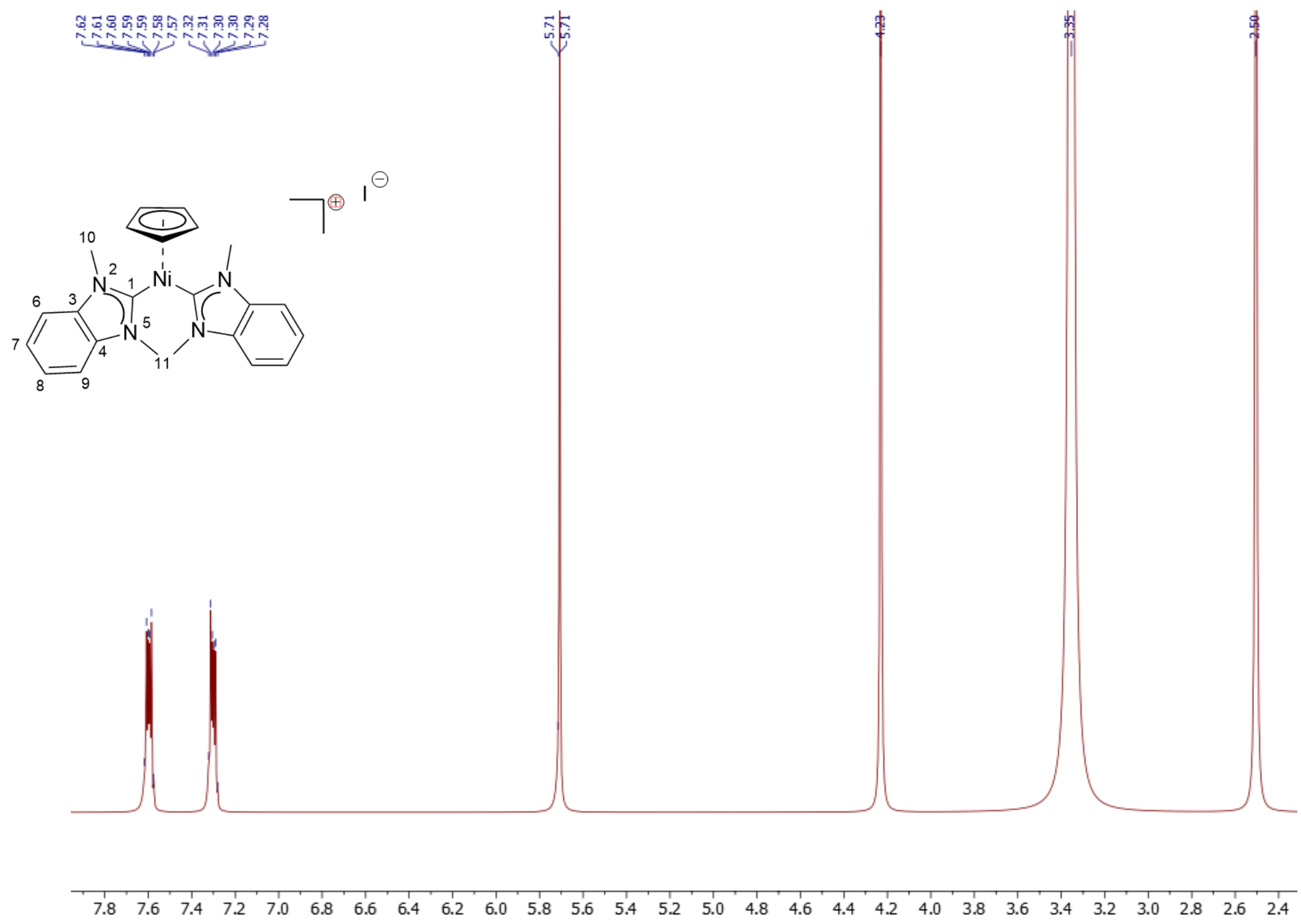

Figure S1. ^{1}H NMR spectrum of complex **8** in DMSO-d_6.

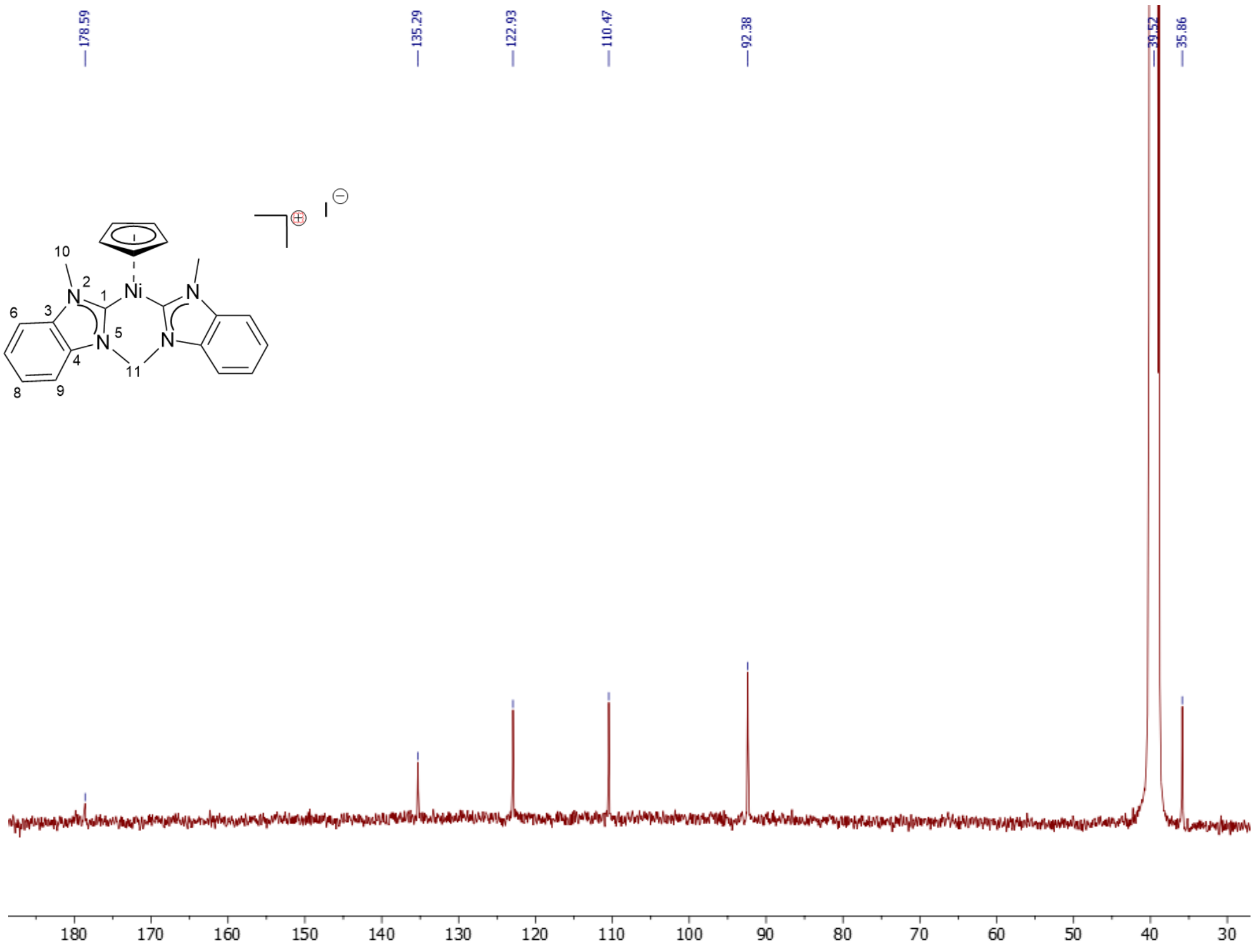

Figure S2. $^{13}C\{^{1}H\}$ NMR spectrum of complex **8** in DMSO-d_6.

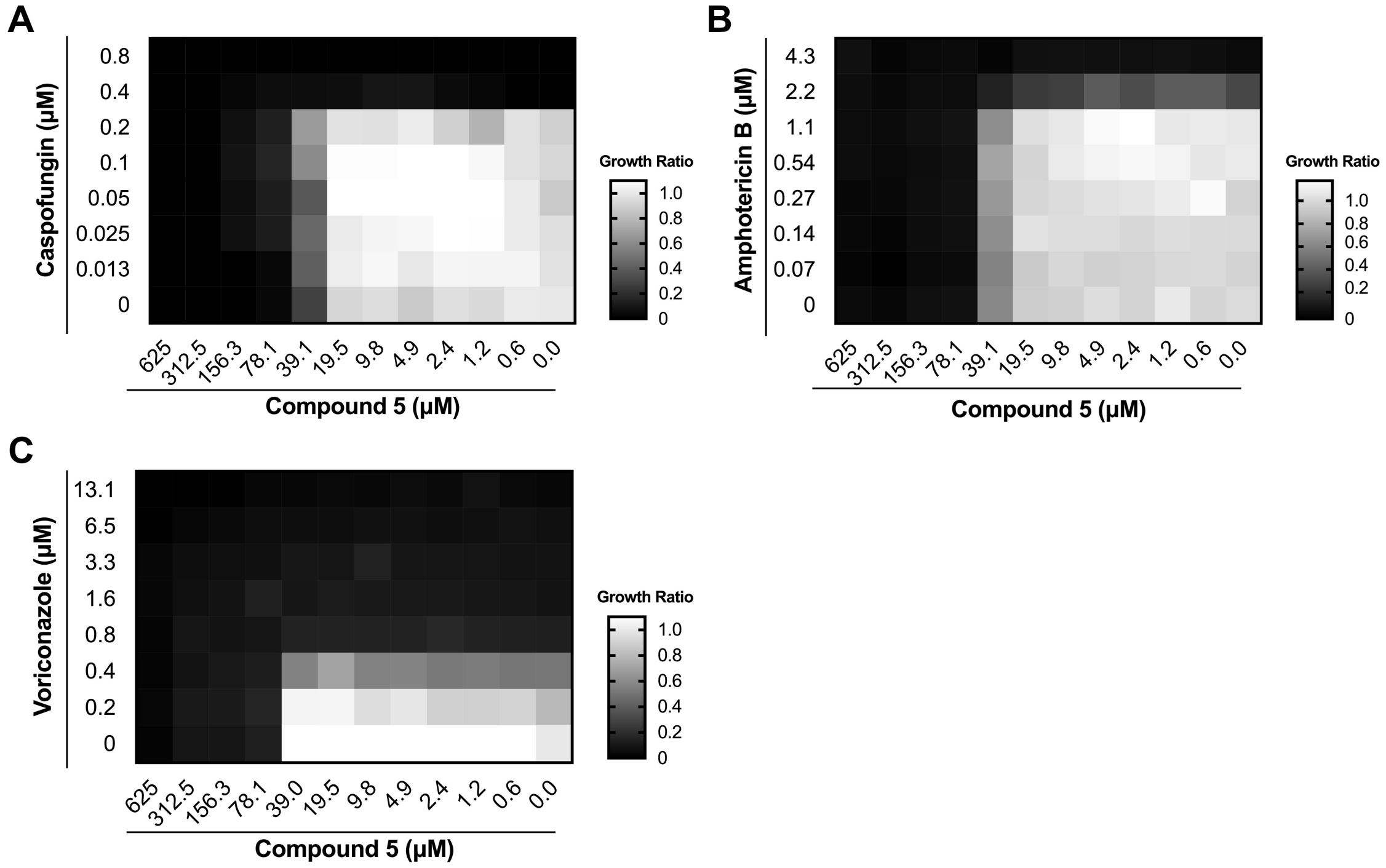

Figure S3. Compound 5 does not synergistically interact with other antifungal drugs. Checkerboard assays were performed to evaluate the growth of *N. glabratus* in medium supplemented with compound 5 combined with caspofungin (A), amphotericin B (B) or voriconazole (C). After 24 h, OD_{600} was measured, values were normalized to untreated controls.

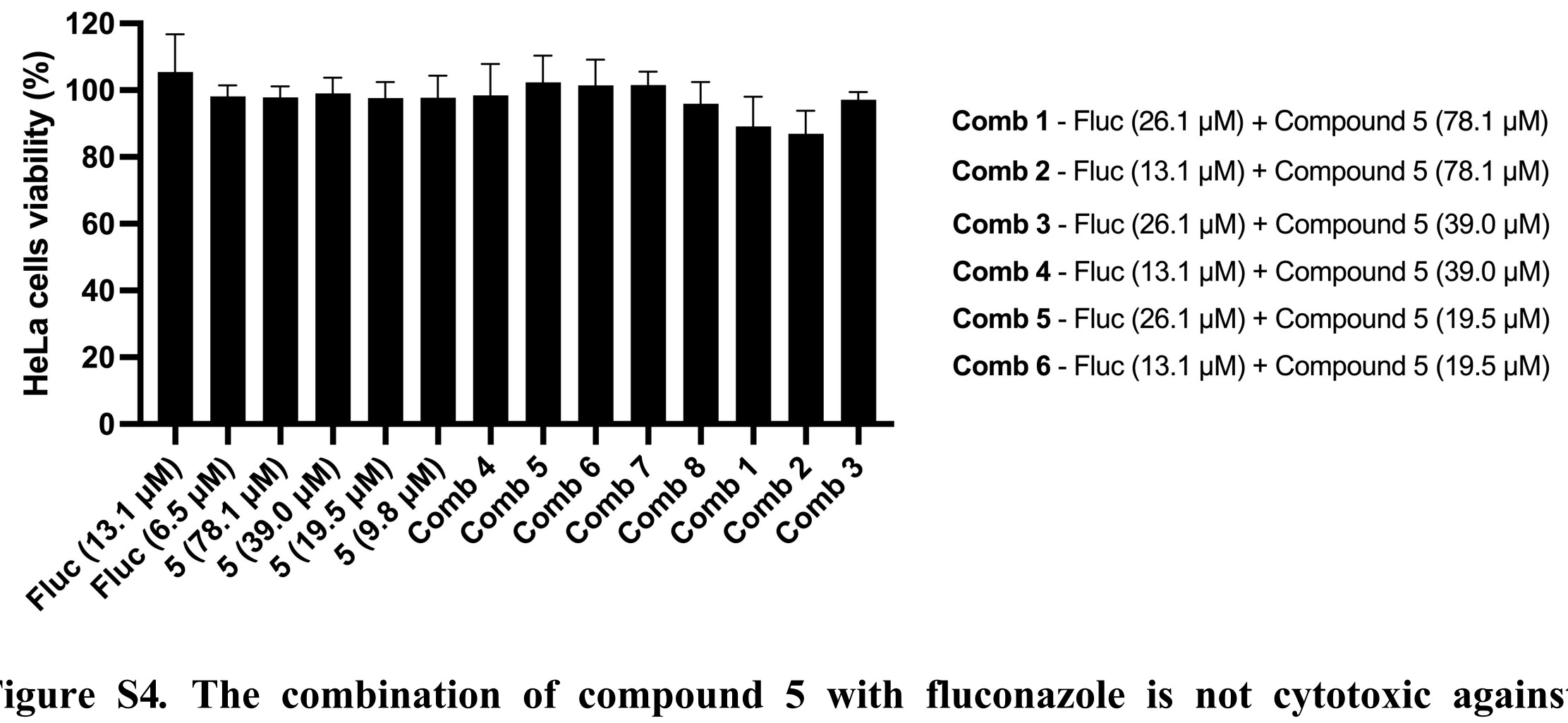

Figure S4. The combination of compound 5 with fluconazole is not cytotoxic against mammalian cells. Cellular viability after treatment with the indicated concentrations of fluconazole (Fluc), compound 5 (5) or their combination (Comb) was evaluated using the MTT assay and the values are expressed as the percentage of viability relative to the untreated control.

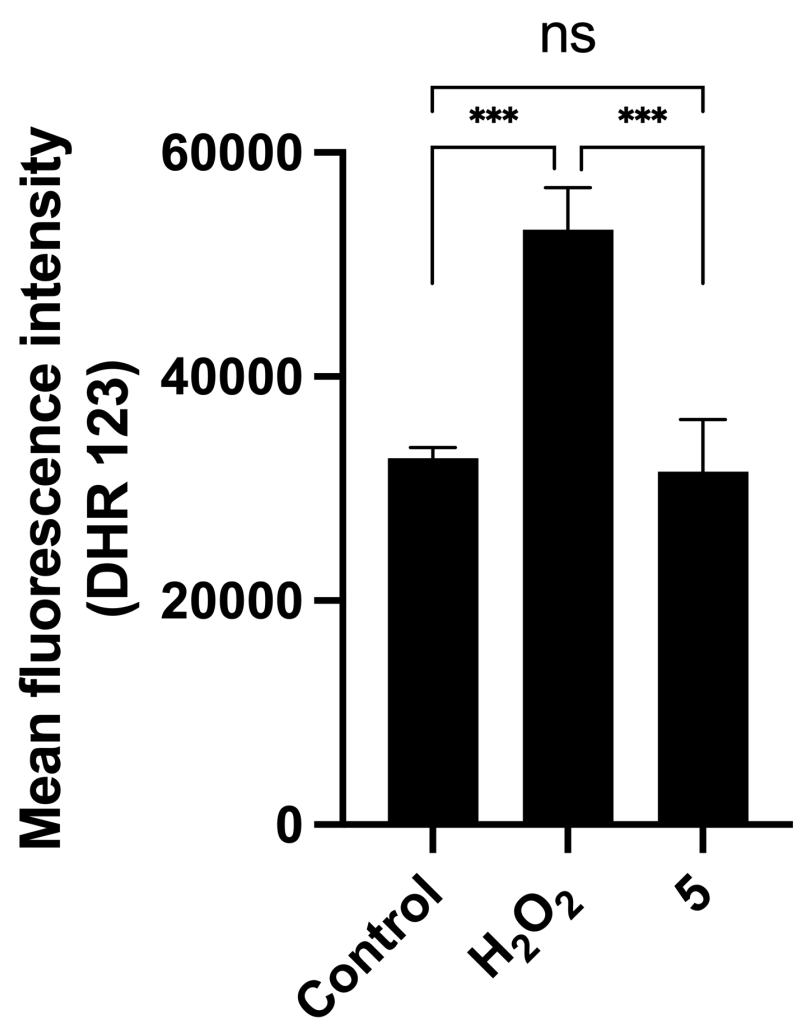

Figure S5. Compound 5 does not induce reactive oxygen species. Intracellular ROS levels in *N. glabratus* cells, untreated (Control) or treated with 10 mM hydrogen peroxide (H_2O_2) or 78.1 µM compound 5 (5), were quantified using the fluorescence dye dihydrorhodamine 123 (DHR 123). Statistical significance was assessed using one-way ANOVA with Tukey's HSD post hoc test (***p<0.001).
